# Unravelling the skillset of point-of-care ultrasound: a systematic review

**DOI:** 10.1186/s13089-023-00319-4

**Published:** 2023-04-19

**Authors:** Tessa A. Mulder, Tim van de Velde, Eveline Dokter, Bas Boekestijn, Tycho J. Olgers, Martijn P. Bauer, Beerend P. Hierck

**Affiliations:** 1 Department of Internal Medicine, Leiden University Medical Center, Leiden, The Netherlands; 2 Department of Neuropsychology, Leiden University, Leiden, The Netherlands; 3 Department of Radiology, Leiden University Medical Center, Leiden, The Netherlands; 4 Department of Internal Medicine, University Medical Center Groningen, Groningen, The Netherlands; 5 Department of Anatomy and Embryology, Leiden University Medical Center, Leiden, The Netherlands; 6 Center for Innovation of Medical Education, Leiden University Medical Center, Leiden, The Netherlands; 7 Department of Clinical Sciences-Anatomy and Physiology, Veterinary Medicine Faculty, Utrecht University, Utrecht, The Netherlands

**Keywords:** Point-of-care-ultrasound, Training, Visuospatial, Psychomotor, Knowledge, Competence, Skill, Ability

## Abstract

**Background:**

The increasing number of physicians that are trained in point-of-care ultrasound (POCUS) warrants critical evaluation and improvement of current training methods. Performing POCUS is a complex task and it is unknown which (neuro)cognitive mechanisms are most important in competence development of this skill. This systematic review was conducted to identify determinants of POCUS competence development that can be used to optimize POCUS training.

**Methods:**

PubMed, Web of Science, Cochrane Library, Emcare, PsycINFO and ERIC databases were searched for studies measuring ultrasound (US) skills and aptitude. The papers were divided into three categories: “Relevant knowledge”, “Psychomotor ability” and ‘Visuospatial ability’. The ‘Relevant knowledge’ category was further subdivided in ‘image interpretation’, ‘technical aspects’ and ‘general cognitive abilities’. Visuospatial ability was subdivided in visuospatial subcategories based on the Cattell-Horn-Carroll (CHC) Model of Intelligence v2.2, which includes visuospatial manipulation and visuospatial perception. Post-hoc, a meta-analysis was performed to calculate pooled correlations.

**Results:**

26 papers were selected for inclusion in the review. 15 reported on *relevant knowledge* with a pooled coefficient of determination of 0.26. Four papers reported on *psychomotor abilities,* one reported a significant relationship with POCUS competence. 13 papers reported on *visuospatial abilities*, the pooled coefficient of determination was 0.16.

**Conclusion:**

There was a lot of heterogeneity in methods to assess possible determinants of POCUS competence and POCUS competence acquisition. This makes it difficult to draw strong conclusions on which determinants should be part of a framework to improve POCUS education. However, we identified two determinants of POCUS competence development: relevant knowledge and visuospatial ability. The content of *relevant knowledge* could not be retrieved in more depth. For visuospatial ability we used the CHC model as theoretical framework to analyze this skill. We could not point out psychomotor ability as a determinant of POCUS competence.

**Supplementary Information:**

The online version contains supplementary material available at 10.1186/s13089-023-00319-4.

## Background

The number of physicians that are trained in point-of-care ultrasound (POCUS) is growing. POCUS is defined as the use of portable ultrasonography (US) at the patient’s bedside, which is performed and interpreted instantaneously [1, 2]. The utility of POCUS depends on the experience and skill of the operator and, therefore, proper training and assessment of competence are crucial [3]. As more and more US training and assessment methods are being developed, differences in both training and assessment methods become more apparent [4–6]. With the increasing number of physicians that are required to learn POCUS, there is a need for critical evaluation of the methods used to teach POCUS, as well as the methods used to assess competence and competence development. Current educational US literature mainly focuses on the overall effects of US courses, with the assessment of performance before and after training, often leading to positive results. The underlying mechanism on which this process of improvement is based, i.e. the set of (cognitive) factors that predict efficient POCUS competence development, is often approached as a black box and therefore remains largely unknown.

Performing and interpreting US examination comprises a unique and complex set of actions. The operator must be familiar with ultrasonography physics and must have sufficient knowledge of regional 3-dimensional (3D) anatomy and pathophysiology. That can be a challenge, since the US screen displays the image in 2D and the operator needs to construct and manipulate a mental 3D representation. The anatomy changes by applying pressure with the probe, and factors like breathing, bowel contents, excess of subcutaneous and visceral adipose tissue, anatomical variations and pathology must be considered to create an adequate image. US is unique compared to other imaging modalities in its operator dependency, requiring correct manipulation of the probe and various US parameters to achieve good image quality.

POCUS competence incorporates a unique combination of skills perceived to include, beside adequate knowledge, various (neuro)cognitive mechanisms, like visuospatial abilities, and psychomotor abilities [6–8]. From other complex medical skills, like laparoscopy and arthroscopy, we know that visuospatial and psychomotor ability are predictors of competence achievement in that field [6, 9, 10]. Visuospatial ability is the capability to generate, transform, and retain structured visual images. That is, to mentally manipulate two-dimensional and three-dimensional figures [11]. Dimensions of visuospatial ability are visuospatial perception and visuospatial manipulation. Visuospatial perception refers to the ability to appropriately perceive the physical location of an object in relation to one’s own body and to identify the physical relationship between different objects. Concretely, in US visuospatial perception describes the interpretation of size, shape, position and motion of organs [12]. Visuospatial manipulation is the ability to perceive complex patterns and mentally simulate how they might look when transformed (e.g. rotated, changed in size, partially obscured, and so forth). This is often tested with the mental rotation test (MRT) in which a more simple object-based transformation is performed [13]. Psychomotor ability means performing motor tasks with exactitude and dexterity, for example, using manual- and finger dexterity and hand–eye coordination while handling a probe [14]. There are various validated tests to assess different domains of visuospatial ability as well as psychomotor skills [15–22]. Relevant knowledge can be measured in several ways. Multiple-choice tests can be designed to measure trainee knowledge of ultrasound physics, while image interpretation of still images or short videos can be utilized to evaluate knowledge of anatomy or the recognition of pathology [6, 23]. Furthermore, it is known that a set of other general cognitive abilities are needed to successfully learn a new skill, including for example general reasoning [24, 25]. We hypothesize that this also applies to learning POCUS. The question arises whether there are relevant determinants, like knowledge, psychomotor ability, visuospatial ability and others, of POCUS competence development for POCUS practitioners, and if so, to what extent.

This systematic review summarizes current knowledge on determinants of POCUS competence and competence development in order to identify the framework of skills needed to develop and improve POCUS competence.

## Methods

This protocol (ID 239322) is available for review at the PROSPERO website (https://www.crd.york.ac.uk/PROSPERO/). The review was conducted and reported according to PRISMA standards of quality [26, 27].

### Information sources and search strategy

**Fig. 1:**
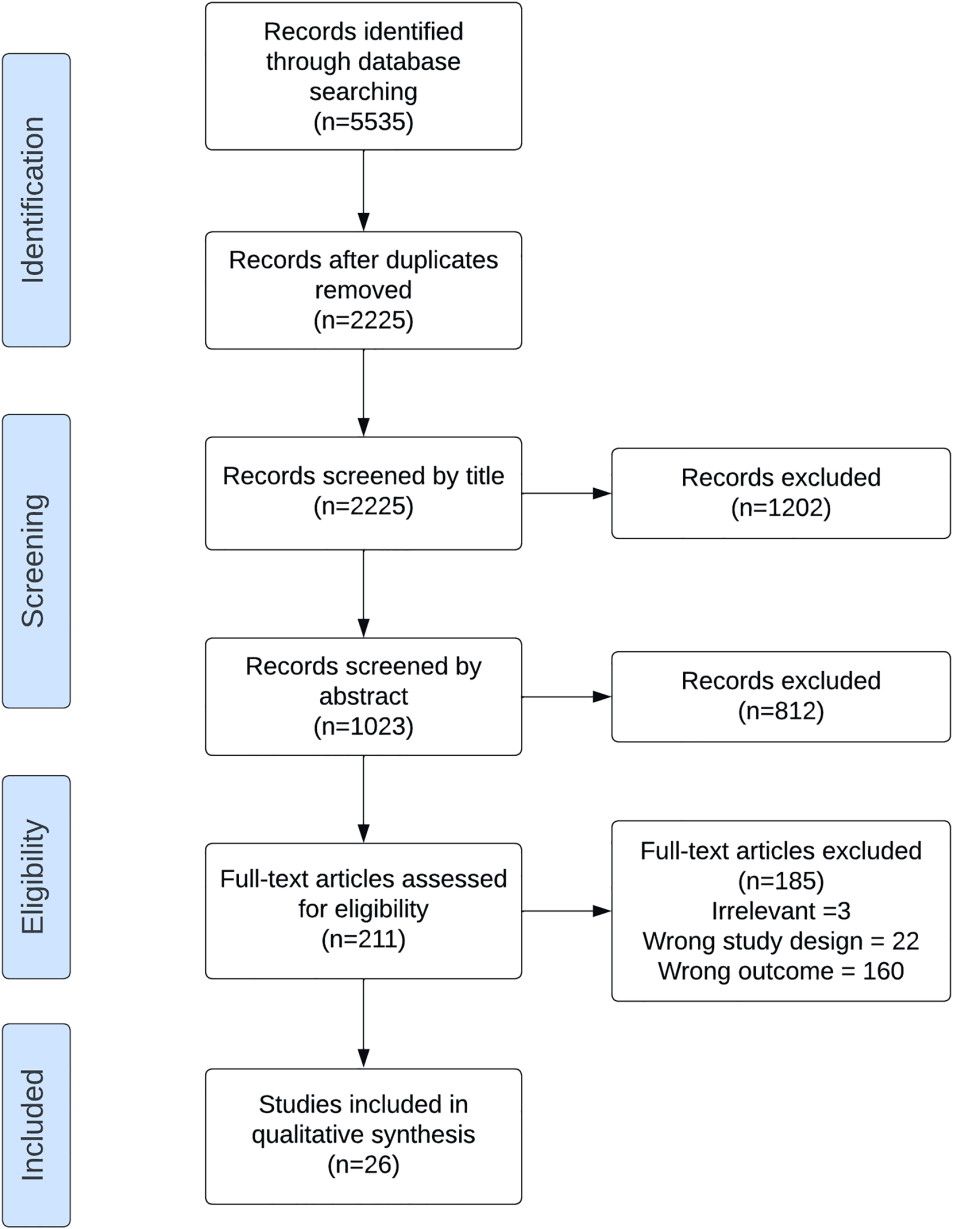
Flow diagram of study selection

PubMed, Web of Science, Cochrane Library, Emcare, PsycINFO and ERIC databases were searched for studies measuring US skills and abilities on 5 March 2021. (Fig. 1) The entire search strategy can be found in Additional file 1: Appendix S1.

### Eligibility criteria and study selection

The following criteria were used to assess the eligibility of studies found by the search strategy: the study has to be an original, full peer-reviewed paper written in English, the study must be either an observational or interventional clinical trial, which includes an objective measurement of specific skills and a description or calculation of a relationship with US performance and the study subjects must be studying or working in the medical field. We excluded clinical trials with self-reported measurements of skills, conference papers, meeting abstracts, letters to the editors, reviews, meta-analyses, comments, and study protocols.

Two independent authors (TM and TV) screened all titles and abstracts in duplicate and excluded clearly irrelevant studies. The remaining articles underwent an independent, full-text screening by the same authors in duplicate. Conflicts during the selection process were resolved by a third reviewer (BH).

### Data extraction

Data from eligible studies were extracted using an extraction sheet. Data items extracted are as follows: number of participants, description of participants, baseline measurements before training or intervention regarding either POCUS competence and/or competence determinants, post-intervention scores regarding POCUS competence and correlations between used interventions and POCUS competence.

### Risk of bias in individual studies

To assess methodological quality in individual studies, the Medical Education Research Study Quality instrument (MERSQI) was used by the two authors independently [28]. This tool has been validated for medical education [29]. The tool has 18 points in 6 domains: study design, sampling, data type, validity, analysis, and outcomes. Furthermore, we assessed validity of possible determinants and outcome measures using the Messick framework [30].

### Data analysis

Descriptive analysis was used to summarize the included studies and to describe the effects of various skills on US performance. The papers were divided into three categories based on the skills that were measured during the studies. These categories were: “Relevant knowledge”, “Psychomotor ability” and ‘Visuospatial ability”. The ‘Relevant knowledge’ category was further subdivided in ‘image interpretation’, ‘technical aspects’ and ‘general cognitive abilities’. Visuospatial ability was further focused on visuospatial manipulation and visuospatial perception. The visuospatial manipulation category entails all the tests which primarily measure mental manipulation of (limited) visual information. The visuospatial perception category entails all the tests which primarily measure perceptual accuracy.

As we did not encounter sufficient studies of consistent design and quality, a formal meta-analysis was not feasible. However, when studies reported an *R*^2^ statistic, this was taken into account when analyzing variance explained by the relevant determinants. If a study did not report an *R*^2^, linearity of data was assessed and, if applicable, *R*^2^ was calculated ad hoc for the purpose of this review. Furthermore, after examination of the papers a decision was made to calculate pooled correlations, to gain more insight in how certain tests and domains relate to the ability to learn and perform US. Using the *metacor* function from the *meta* package in R version 4.1.3 all correlations underwent Fisher’s Z transformation and were then pooled using the random-effects model [31]. These pooled correlations were then squared to obtain a pooled coefficient of determination. The determination coefficient reports how much variance of a dependent variable is explained by a determinant [32]. The random-effects model was applied because during examination of the papers, heterogeneity (using Cochran’s Q) of multiple kinds, e.g. differences in study samples and test instruments, was found. This makes the fixed-effects model inappropriate to calculate the pooled effect size. This was done for both the complete set of reports/studies, as well as separately for the studies in the knowledge and visuospatial domains. This could not be done for the psychomotor domain since no correlations were reported in those studies.

## Results

### Study selection

The applied search strategy yielded a total of 5535 potentially relevant papers. Removing duplicates and then screening both titles and abstracts resulted in the removal of 5324 papers. The remaining 211 papers were screened in full text, and a final 26 papers were selected for inclusion in the review. This process is highlighted in Fig. 1. There was a substantial agreement between both authors (TM and TV) during the study selection. Both during title and abstract screening (Cohen’s kappa 0.65) and during the full text selection (Cohen’s kappa 0.65). A large number of studies in the full text screening section appeared eligible at first, but at closer inspection lacked meaningful analysis regarding the relationship between measured variables and the ability to learn and/or assess US competence.

### Study characteristics

Of the 26 studies, 15 reported on the relationship between relevant knowledge and US competence, three studies reported on the relationship between measured psychomotor ability and (gaining) US competence and a total of 13 papers reported on the relationship between visuospatial ability measurements and (gaining) US competence. The papers spanned various US domains; general US, sonography for trauma (FAST), musculoskeletal US, transthoracic echocardiography, US-guided central venous access, obstetric US, brachial plexus US, US-guided regional anaesthesia (UGRA), and ultrasonography for veterinary students. US competence was assessed on standardized patients, volunteers, bench models, simulators, or turkey breasts. Various competence measures were used to assess ultrasound skill level. OSCE scores of performing ultrasound on a standardized patients were mostly used (*n* = 11). Furthermore, time of completion of the ultrasound task and image interpretation of live images during an ultrasound examination were used. Few studies tried to identify determinants by looking at differences of those determinants between novice and expert ultra-sonographers. Validity evidence was not found for all measures used. Most assessment methods were validated in terms of content and relationships with other variables. See Additional file 1: Appendix S2 for all study characteristics.

### Study appraisal

To assess risk of bias for each study the MERSQI was used. The lowest score attained on the MERSQI was a 10, while a 15.5 was the highest score. There was a median score of 12.5 across all included studies. MERSQI scores can be found in Additional file 1: Appendix S3.

### Relevant knowledge

**Fig. 2:**
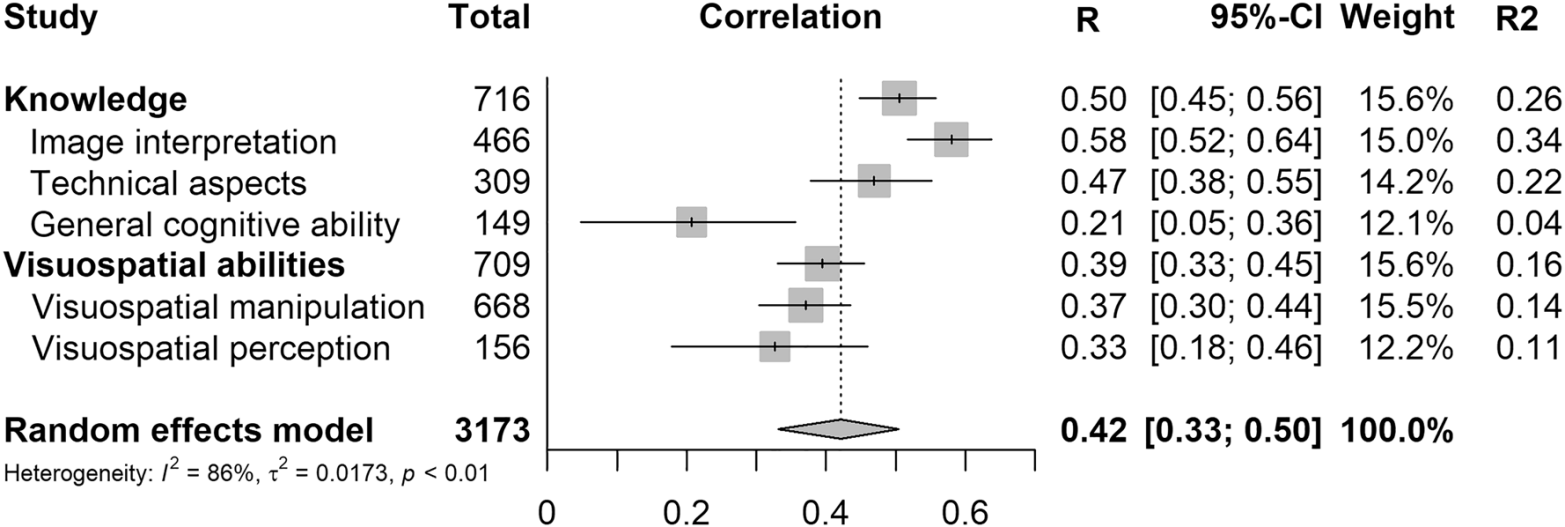
Forest plot of the pooled correlations of the included studies divided by cognitive domain. *R* *correlation coefficient. 95% CI* *95% Confidence Interval. R2* *determination coefficient.* The pointed line represents the pooled correlation of the random effects model of all included studies, the grey box represents the weight of the studies. I^2^ fraction of variance due to heterogeneity. T^2^ the estimated standard deviation of underlying effects across the studies

From the 15 papers reporting on the relationship between relevant knowledge and the ability to learn or perform US, eight reported a significant relationship between at least one of their measured variables and the ability to learn or perform US. [33–40] The studies describing these relationships covered various medical domains (Table 1). The significant associations were found in FAST, musculoskeletal US training for rheumatology fellows, transthoracic echocardiography, US-guided central venous access and general US education, as well as in US education in low to middle income countries [33–40]. Relevant knowledge was tested with various multiple-choice tests, mostly containing questions about US physics, knobology, image interpretation and basic anatomical knowledge. 11 studies tested relevant knowledge by means of image interpretation, 6 studies used questions about technical aspects of US (knobology, US physics). Furthermore, five papers looked at the relationship between general reasoning, memory and cue utilization (the application of cue-based associations retrieved from memory), and US performance, of which Berman et al. [34] looked at general reasoning scores using the Kit of Factor Referenced Cognitive tests. Two of the 15 papers reported a determination coefficient. Stolz et al. [41] reported this to describe the relationship between baseline US knowledge, consisting of basic US physics, system workflow, and anatomy, ability to recognize anomalies, appropriate US settings, and US competence. With an R^2^ of 0.028 they state that their written pre-test is not a good predictor of US interpretation ability. On the other hand, Schott [39] reported a much higher determination coefficient of 0.60 between their knowledge test and POCUS competence. For all other papers reporting a correlation statistic, primarily Pearson correlation and Spearman’s rho, *R*^2^ was calculated, see Table 2. The knowledge domain had a pooled correlation value of *r* = 0.51, *p* ≤ 0.0001. This correlation equates to a coefficient of determination of 0.26. This implies that roughly 26% of the ability to learn and or perform US in these papers is attributed to relevant knowledge. See Fig. 2. When the knowledge domain was assessed for heterogeneity, Cochran’s Q was 73.96, *p* ≤ 0.0001.

**Table 1:** Summary of included studies divided by cognitive domain with pooled correlation and determination coefficient

Domain	Articles	Tests used	Sample size (total)	Pooled correlation [95% CI]	Determination coefficient
Knowledge			*k* = 13 *n* = 716	0.51 [0.35; 0.63] *p* < 0.0001	0.26
Image interpretation	Bell et al. [33], Chung et al. [35], Janjigian et al. [36], Kissin et al. [37], Nielsen et al. [38], Schott et al. [39], Sisley et al. [42], Stolz et al. [41], Woodworth et al. [43]	Different multiple choice and written tests	*k* = 9 *n* = 466	0.58 [0.43; 0.70] *p* < 0.0001	0.34
Technical aspects	Janjigian et al. [31], Nielsen et al. [33], Schott et al. [34], Sisley et al. [44], Stolz et al. [45] Werner et al. [46]	Different multiple choice and written tests	k = 6 *n* = 309	0.47 [0.20; 0.67] *p* = 0.0013	0.22
General cognitive ability	Berman et al. [34], Carrigan et al. [44], Shafqat et al. [47]	Kit of Factor Reference Cognitive tests, EXPERTise 2.0 Echocardiography edition, Alice Heim Group Ability Test, Numerical Reasoning Test, Digit Symbol Substitution Test	*k* = 2 *n* = 149	0.21 [− 0.09; 0.47] *p* = 0.1741	0.04
Psychomotor ability					
Psychomotor ability	Chapman et al. [45], Dromey et al. [46], Smith et al. [48], Walker et al. [49]	Crawford small parts dexterity test, Dimensionless Squared Jerk, Project image test (ZigZag test), Purdue Pegboard test, Semmes–Weinstein Monofilament Sensory Test	*k* = 4 *n* = 120	No correlation reported	
Visuospatial ability			*k* = 8 *n* = 708	0.39 [0.23; 0.54] *p* < 0.0001	0.16
Visuospatial manipulation			k = 7 *n* = 668	0.37 [0.17; 0.54] *p* = 0.0005	0.14
Visualization	Berman et al. [34], Clem et al. [51], Clem et al. [46], Chapman et al. [45]	Kit of Factor Reference Cognitive Tests (spatial orientation and visualization subtests), Revised Minnesota Paper Form Board Test, Surface Development Test	*k* = 3 *n* = 179	0.39 [0.09; 0.63] *p* = 0.0116	0.15
Mental rotation	Chapman et al. [45], Chuan et al. [52], Duce et al. [53], Frederiksen et al. [54], Hewson et al. [55], Shafqat et al. [47], Miller et al. [56], Walker et al. [49]	Mental Rotation test, Revised Vanderberg and Kruse Mental Rotation Test A	*k* = 4 *n* = 489	0.38 [0.0864; 0.61] *p* = 0.0125	0.15
Visuospatial perception			*k* = 3 *n* = 156	0.33 [0.14; 0.49] *p* = 0.0009	0.11
Lower order perception	Smith et al. [48]	Pelli-Robson contrast acuity testing	*k* = 1 *n* = 40	No correlation reported	
Closure speed	Chapman et al. [45], Duce et al. [53], Smith et al. [48]	Block Design Test (WAIS-IV), Gestalt Completion Test, Matrix reasoning Test, The Snowy Picture Test, Visual puzzles	*k* = 2 *n* = 73	0.43 [0.22; 0.60] *p* = 0.0002	0.19
Flexibility of closure	Berman et al. [34] Chapman et al. [45], Duce et al. [53], Shafqat et al. [47]	Abstract Reasoning Test, Concealed Figures Test, Group Embedded Figures Test, Kit of Factor Reference Cognitive Tests (flexibility of closure subtest)	*k* = 2 *n* = 143	0.14 [− 0.02; 0.30] *p* = 0.0839	0.02
Visual memory	Carrigan et al. [44]	Novel Object Memory Test	*k* = 1 *n* = 39	No correlation reported	
Spatial scanning	Smith et al. [48]	Trail making test (TMT)	*k* = 1 *n* = 40	No correlation reported	

*k*: number of studies included in analysis; *n:* number of participants included in analysis; pooled correlation: the pooled correlation calculated with the random effects model; *95% CI:* 95% confidence interval; *p:* *P*-value

**Table 2:** Calculated and reported determination coefficients of the included studies

Relevant measurement	Is the correlation linear?	Reported *R*^2^	Calculated *R*^2^
Knowledge			
Baker et al. [31]	Yes	–	*R*^2^ = 0.053
Bell et al. [32]	Yes	–	*R*^2^ = 0.194 ***
Berman et al. [50]	Yes	–	*R*^2^ = 0.119 *
Carrigan et al. [44]	No correlation reported	–	–
Chung et al. [35]	Yes	–	*R*^2^ = 0.116 *
Janjigian et al. [36]	Yes	–	*R*^2^ = 0.608 at one-year,****R^2^ = 0.281 on post-two-day assessment*
Kissin et al. [37]	Yes	–	First group: *R*^2^ = 0.49****, second group: *R*^2^ = 0.348 *
Nielsen et al. [38]	No, spearman's rho is nonlinear correlation	–	*R*^2^ = 0.608 for residents****
Schott et al. [39]	Yes	*R*^2^ = 0.60****	
Shafqat et al. [47]	No, spearman's rho is nonlinear correlation		NRT-20: *R*^2^ = 0.0025 AH4: *R*^2^ = 0.0081
Sisley et al. [42]	Yes	–	Precourse: *R*^2^ = 0.04, postcourse *R*^2^ = 0.02
Stolz et al. [41]	Yes	*R*^2^ = 0.028	–
Tolsgaard et al. [57]	No correlation reported	–	–
Werner et al. [40]	No, spearman's rho is nonlinear correlation	–	*R*^2^ = 0.152*
Woodworth et al. [43]	Yes	–	Pretest: *R*^2^ = 0.221, posttest: *R*^2^ = 0.410
Psychomotor ability			
Chapman et al. [45]	No correlation reported	–	–
Dromey et al. [58]	No correlation reported	–	–
Smith et al. [48]	No correlation reported	–	–
Walker et al. [49]	No correlation reported	–	–
Visuospatial ability			
Berman et al. [34]	Yes	–	Flexibility of closure: *R*^2^ = 0.040 Spatial orientation: *R*^2^ = 0.001, Visualization: *R*^2^ = 0.006
Carrigan et al. [44]	No correlation reported	–	–
Chapman et al. [45]	No correlation reported	–	–
Chuan et al. [52]	No correlation reported	–	–
Clem et al. [46]	Yes	*R*^2^ = 0.36**	–
Clem et al. [46]	Yes	After 30 h: *R*^2^ = 0.21*, after two semesters *R*^2^ = 0.23*	–
Duce et al. [53]	No, spearman's rho is a linear correlation	–	Matrix reasoning: *R*^2^ = 0.144*, MRT-A: *R*^2^ = 0.130*
Frederiksen et al. [54]	Yes	–	MRT + global image rating: *R*^2^ = 0.476***, MRT + global image evaluation: *R*^2^ = 0.194*, MRT + probe orientation: *R*^2^ = 0.314***
Hewson et al. [55]	No correlation reported	–	–
Miller et al. [56]	Yes	–	MRT + posttest knowledge scores: *R*^2^ = 0.044****, MRT + PLAX score: *R*^2^ = − 0.16, MRT + PLAX time: *R*^2^ = − 0.04, MRT + hepatorenal score: *R*^2^ = − 0.12, MRT + hepatorenal time: *R*^2^ = − 0.16
Shafqat et al. [38]	No, Spearman’s rho is a nonlinear correlation	–	MRT score *R*^2^ = 0.221***, Group Embedded Figures Test: *R*^2^ = 0.004
Smith et al. [48]	No, Spearman’s rho is a nonlinear correlation	–	Block design test + global ultrasound performance: *R*^2^ = 0.221***

*R*^2^: coefficient of determination; MRT: Mental Rotation Test; GRS: Global Rating Scale; CES: Composite Error Score

*p-value < 0.05, **p-value < 0.02, ***p-value < 0.01, ****p-value < 0.001

### Psychomotor ability

Four papers reported on psychomotor abilities in relation to US performance (Table 1). Psychomotor ability was measured by various tests, i.e. Projected Image Testing (Zig–Zag Test), Purdue Peg Board Test, Crawford Small Parts Dexterity Test, Sennes-Weinstein Monofilament Sensory Testing and the Dimensionless Squared Jerk. One study reported a significant relationship between the Dimensionless Squared Jerk, a validated motion metric that measures deliberate hand movements, and US expertise in obstetric US [58], while the other three papers did not find a significant relationship between psychomotor skills and US competence. [45, 48, 49] No correlation or determination coefficient was reported in the studies. Walker [49] found a regression coefficient of 0.00056 (*p* = 0.580) for the Grooved Pegboard test performed by the non-dominant hand, and of − 0.0013 (*p* = 0.329) when it was executed by the dominant hand, and time to complete an ultrasound guided cystocentesis task.

### Visuospatial ability

A total of 13 papers reported on the relationship between visuospatial ability measurements and US competence (Table 1). Out of these 13 papers, 10 reported a significant relationship between at least one visuospatial ability measurement and US competence. Significant results were found in brachial plexus sonography, transthoracic echocardiography, UGRA, ultrasonography for veterinary students and general ultrasonography. To further narrow down which tests were able to provide good predictions for US competence and why, visuospatial subcategories based on the Cattell-Horn-Carroll (CHC) Model of Intelligence v2.2 were used [59]. This model makes a distinction between 11 different forms of visual processing: visualization, speeded rotation, closure speed, flexibility of closure, visual memory, spatial scanning, serial perceptual integration, length estimation, perceptual illusions, perceptual alterations, and imagery. For a description of these categories, see Table 3. The most frequently used ability test for visuospatial ability was the MRT (*n* = 8). In these 13 papers, 12 tests were done that fit into the visuospatial *manipulation* category. 8 out of these 12 tests were adaptations of the MRT. 4 specified that they used the Revised Vanderberg and Kruse Mental Rotation Test A. Therefore, mental rotation is reported here in its own category to see if this specific test warrants its prominent appearance in US research. A total of 13 tests belonging to the visuospatial *perception* category were used in these papers [12]. See Additional file 1: Appendix S4 for an overview of the different aptitude tests used, divided by main and subcategory. Six papers reported correlation coefficients and two papers a determination coefficient (Table 2). Clem et al. [51] reported that 0.36 of US competence can be predicted by visuospatial ability. The other determination coefficient is reported by another study of Clem et al. [46] They state that 0.23 of US competence can be predicted by spatial ability after two full semesters of instructions. The pooled correlation of the visuospatial domain had a value of *r*(8) = 0.39, *p* ≤ 0.0001. The coefficient of determination was 0.16. This implies that roughly 16% of the ability to learn and or perform US across these studies could be attributed to the measured visuospatial ability. When the visuospatial domain was assessed for heterogeneity, Cochran’s Q was 27.37, *p* = 0.011. The papers using tests in the visuospatial manipulation category had a pooled correlation of r(7) = 0.37, *p* = 0.0005 and a pooled coefficient of determination of 0.14. This implies that roughly 14% of the ability to learn and or perform US across these studies could be attributed to the measured visuospatial manipulation abilities. The papers using tests in the visuospatial perception category had a pooled correlation of r(3) = 0.33, *p* =  < 0.0001 and a pooled coefficient of determination of 0.11. This implies that roughly 11% of the ability to learn and or perform US across these studies could be attributed to the measured visuospatial perception abilities. See Fig. 2. To see if the MRT warrants its prominent position in US research, pooled correlations were also calculated separately for the MRT, compared to the other visuospatial manipulation tests used. All the MRTs combined had a pooled correlation of r(5) = 0.415, *p* ≤ 0.01 and a pooled coefficient of determination of 0.17.

**Table 3:** Cattell–Horn–Carroll (CHC) explanation

Visual processing subgroup	Explanation
Visualization	The ability to perceive complex patterns and mentally simulate how they might look when transformed (e.g., rotated, changed in size, partially obscured, and so forth)
Speeded rotations (spatial relations)	The ability to solve problems quickly using the mental rotation of simple images
Closure speed	Ability to quickly identify a familiar meaningful visual object from incomplete (e.g., vague, partially obscured, disconnected) visual stimuli, without knowing in advance what the object is
Flexibility of closure	Ability to identify a visual figure or pattern embedded in a complex distracting or disguised visual pattern or array, when knowing in advance what the pattern is
Visual memory	Ability to remember complex images over short periods of time (less than 30 s)
Spatial scanning	Ability to visualize a path out of a maze or a field with many obstacles
Serial perceptual integration	Ability to recognize an object after only parts of it are shown in rapid succession
Length estimation	The ability to visually estimate the length of objects
Perceptual illusions	The ability to not be fooled by visual illusions
Perceptual alterations	Consistency in the rate of alternating between different visual perceptions
Imagery	Ability to mentally imagine very vivid images

## Discussion

In this systematic review and meta-analysis, we describe several (neuro)cognitive mechanisms that correlate with the development of POCUS competence. Combined data from various studies revealed relevant knowledge and visuospatial ability as determinants of the ability to acquire POCUS competence. Psychomotor skills have been described in only one study to significantly affect POCUS competence development.

To design effective competency-based skills training programs, it is imperative to determine which underlying mechanisms or skills relate to the acquisition of POCUS competence. In our dataset of 26 papers, only four described a determination coefficient to predict how much variance of US competence could be explained by their measured determinants. [39, 42, 46, 51] Therefore, we decided to use the published data to perform a post-hoc calculation of the determination coefficients of 17 additional studies and found a pooled coefficient of determination of 16%. This implies that 16% of the ability to learn and/or perform US, as measured in these studies, can be attributed to the variables that were reported. These variables could be used to predict learner performance and to finetune personalized and adaptive education in the future. This is important as a systematic review and meta-analysis by Fontaine et al. [62] describes that adaptive e-learning environments have improved learning outcomes on both knowledge and practical skills compared to traditional methods of education and training.

Relevant knowledge is a nonspecific term and the type of knowledge that is actually relevant for POCUS competence development cannot be easily distinguished. Despite that, the pooled coefficient of determination for the knowledge domain implies that roughly 26% of the ability to learn and/or perform US might be attributed to relevant forms of knowledge. In many studies, both anatomical knowledge and image interpretation are used as outcome measures, but not all studies describe significant relationships between these types of knowledge and POCUS competence development. The fact that not all studies found significant relations is probably due to the lack of standardized tests for assessing both knowledge and POCUS competence. As expected, many studies identified relationships between POCUS competence development and pre-existing knowledge about technical aspects of ultrasound. However, since all studies used multiple-choice tests to assess various aspects of knowledge, we cannot distinguish the contribution of pre-existing technical knowledge from the other types of knowledge. When looking at general cognitive abilities, e.g. among others the capacity to acquire knowledge and competence, results are equivocal. No correlations were found between POCUS competence development and the numerical reasoning test (testing fluid intelligence, abstract reasoning, and problem-solving) or Alice Heim Group Ability test (verbal, mathematical, and spatial reasoning). [44, 47] And although Berman et al. [34], using a paper-and-pencil test, describe a correlation between general reasoning and POCUS competence development, Shafqat et al., [47] could not find such a relationship using a validated score of a UGRA task. Apparently, both tests measured different aspects of cognitive ability, and therefore one can only draw conclusions about the relation between POCUS competence development and a specific test score rather than drawing conclusions about underlying cognitive mechanisms in general.

Various tests are available to measure aspects of visuospatial ability. When looking at pooled correlations between the visuospatial *manipulation* and visuospatial *perception* categories, the visuospatial manipulation category appears to be more correlated with the ability to learn and or perform US (coefficient of determination of 14% vs 11%). Although this is only a slight difference, the skill to mentally transform and rotate the image of e.g. an organ is possibly a more important determinant than the mere observational ability to perceive and visually understand spatial information such as shapes, positions, and motions. [12] While high MRT test scores often relate to high POCUS competence levels (see Table 1) others, like the snowy picture test do not. As visuospatial ability inherits various aspects of spatial cognition, like mental rotation and transformation [63, 64], the ability to mentally rotate objects may be more relevant for US performance than the ability to quickly identify a familiar visual object from incomplete visual stimuli. When focussing on the other aspects of the CHC model, studies reported correlations with closure speed and flexibility of closure [34, 47, 48, 53], but no correlations were reported for the other perception subcategories. For this reason, no meaningful analysis can be done on which perception subcategories are more relevant than others. In addition, it remains difficult to draw any conclusions about the precise cognitive skill(s) that is/are responsible for modifying POCUS competence development, as the aptitude tests usually cover more than one skill. The underlying framework of visuospatial ability can be used in various ways to improve US education. Chuan et al. [52] showed that if medical students with low visuospatial ability receive extra training in mental rotation, they can achieve the same UGRA performance scores as their fellow students with higher visuospatial abilities. Furthermore, Hewson et al. [55] specifically trained students’ mental rotation with a simple task and improved UGRA performance. Although UGRA is probably a more complex skill than non-interventional POCUS, visuospatial skills also contribute to non-interventional US performance. [46, 53, 54, 56].

Less insight was gained into the relationship with psychomotor ability. Within our dataset, only Dromey et al. [58] described a relation between Dimensionless Squared Jerk scores and POCUS competence. Dimensionless Squared Jerk is a measure of deliberate hand movements and is often used as a measure for psychomotor skills. [65] However, when measured while performing US it will also depend on US competence and cannot be used anymore as a unique measure for psychomotor skills. When it comes to the assessment of other skills, various tests do not clearly distinguish between e.g. visuospatial ability and psychomotor skills, like the Block Design Test and the Digit Symbol Substitution test [17, 18]. Therefore, the psychomotor ability could play a more prominent role than the current literature suggests.

Our findings suggest that it may be beneficial to adjust training based on student characteristics. In our experience, students that fail the POCUS exams are often advised to simply practice more. However, it is known that complex skills are easier to learn if broken down into component skills. [66] Thus, it is conceivable that by identifying a student’s weaker points beforehand and by training this specific shortcoming isolated from the whole complex POCUS skill, the learning curve may steepen. Not only cognitive load may be decreased in an isolated task, but it is also plausible that a specific skill can be taught better and faster in a task specifically designed for that purpose. [67, 68] This skill training does not necessarily have to be integrated into an ultrasound task, but could also be trained in an alternative way. [69].

### Limitations

Limitations can be subdivided into limitations of the included studies and limitations of this systematic review and meta-analysis.

Considering the included studies, one of the major issues in interpreting their results and attempting to construct a framework based on their measurements, is the large amount of heterogeneity among the test instruments used to measure determinants of POCUS competence as well as measuring POCUS competence itself. Moreover, validity evidence was not equivalent for all tests, which added to the difficulty in interpreting the data. A second limitation in some of the studies include the use of cross-sectional design in assessing for the relationship between determinants and competence. Therefore, we cannot be sure if these determinants predict competence. A third limitation is the lack of POCUS-specific papers. For example, US combined with an intervention such as UGRA might provide different outcomes than specific POCUS-focused studies because of e.g. the added complexity of the anaesthesia tasks, especially in the psychomotor domain.

Considering the current study, while the papers found in this systematic review give new insight into the underlying mechanisms of gaining POCUS competence, these mechanisms are unlikely to be solely responsible for the way someone gains POCUS competence. Although we decided, based on an extensive literature search on learning ultrasound skills, to stratify the results into the categories mentioned earlier, our categories may be incomplete. Secondly, when calculating pooled correlations for relevant knowledge and visuospatial skills, many papers did not report correlations or selectively only reported significant correlations. Therefore, pooled correlations should be interpreted with caution. Finally, to construct a framework in which evidence-based variables are used to improve training, or assessment for US competence, a proper understanding of underlying factors is required. Thus, more standardized research needs to be done, with a clear definition of determinant variables, how to measure these, and methods of assessing US competence.

## Conclusion

We identified two determinants of POCUS competence development: relevant knowledge and visuospatial ability. The content of *relevant knowledge* could not be retrieved in more depth. For visuospatial ability we used the CHC model as a theoretical framework to analyze this skill. We could not point out psychomotor ability as a determinant of POCUS competence. The heterogeneity of results makes it difficult to draw strong conclusions about what should and should not be part of a framework used to improve POCUS education and assessment.

## Supplementary Information

**Additional file 1: Appendix S1.** Search strategy. **Appendix S2.** Study characteristics. **Appendix S3.** MERSQI table. **Appendix S4.** Aptitude tests.

## Data Availability

All data are available upon request.

## Abbreviations

3D: 3-Dimensional
FAST: Focused assessment with sonography in trauma
MERSQI: Medical education research study quality instrument
MRT: Mental rotation test
OSCE: Objective structured clinical examination
POCUS: Point-of-care ultrasound
UGRA: Ultrasound guided regional anaesthesia
US: Ultrasonography
