# Supplementary material for: Unravelling the skillset of point-of-care ultrasound: a systematic review
Source: Ultrasound J. 2023 Apr 19;15:19. doi: 10.1186/s13089-023-00319-4 (PMC10115919; doi:10.1186/s13089-023-00319-4)
Supplement: Supplementary file 1 — Additional file 1: Appendix S1. Search strategy. Appendix S2. Study characteristics. Appendix S3. MERSQI table. Appendix S4. Aptitude tests. [file 13089_2023_319_MOESM1_ESM.docx]

**Appendix S1. Search strategy**

**PubMed**

**(**(("Ultrasonography"[majr] OR "Ultrasonics"[majr] OR "ultrasonography"[ti] OR "ultrasound"[ti] OR "ultras*"[ti] OR "sonography"[ti] OR "sonogra*"[ti] OR "echography"[ti] OR "echogra*"[ti] OR "echo"[ti]) AND ("Space Perception"[Mesh] OR "Space Perception"[tw] OR "visuospatial skills"[tw] OR "visuospatial skill"[tw] OR "mental rotation"[tw] OR "perspective taking"[tw] OR "spatial perception"[tw] OR "Psychomotor Performance"[Mesh] OR "psychomotor skills"[tw] OR "psychomotor skill"[tw] OR "Motor Skills"[mesh] OR "motor skills"[tw] OR "motor skill"[tw] OR "dexterity"[tw] OR "visuomotor skills"[tw] OR "visuomotor skill"[tw] OR "Visual Perception"[mesh] OR "anatomical knowledge"[tw] OR "anatomy knowledge"[tw] OR "physiological knowledge"[tw] OR "physiology knowledge"[tw] OR "Spatial Navigation"[mesh] OR "spatial skills"[tw] OR "spatial skill"[tw] OR "visuospatial abilities"[tw] OR "visuospatial ability"[tw] OR "psychomotor abilities"[tw] OR "psychomotor ability"[tw] OR "motor abilities"[tw] OR "motor ability"[tw] OR "visuomotor abilities"[tw] OR "visuomotor ability"[tw] OR "spatial abilities"[tw] OR "spatial ability"[tw] OR "Task Performance and Analysis"[mesh] OR "skills"[tw] OR "skill"[tw] OR "skilled"[tw]) AND ("training"[tw] OR "train*"[tw] OR "Learning"[mesh] OR "learning"[tw] OR "learn*"[tw] OR "Education"[mesh] OR "education"[subheading] OR "educat*"[tw] OR "Students"[mesh] OR "practice"[tw])) **OR** (("Ultrasonography"[Mesh] OR "Ultrasonics"[mesh] OR "ultrasonography"[tw] OR "ultrasound"[tw] OR "ultras*"[tw] OR "sonography"[tw] OR "sonogra*"[tw] OR "echography"[tw] OR "echogra*"[tw] OR "echo"[tw]) AND ("Space Perception"[majr] OR "Space Perception"[ti] OR "visuospatial skills"[ti] OR "visuospatial skill"[ti] OR "mental rotation"[ti] OR "perspective taking"[ti] OR "spatial perception"[ti] OR "Psychomotor Performance"[majr] OR "psychomotor skills"[ti] OR "psychomotor skill"[ti] OR "Motor Skills"[majr] OR "motor skills"[ti] OR "motor skill"[ti] OR "dexterity"[ti] OR "visuomotor skills"[ti] OR "visuomotor skill"[ti] OR "Visual Perception"[majr] OR "anatomical knowledge"[ti] OR "anatomy knowledge"[ti] OR "physiological knowledge"[ti] OR "physiology knowledge"[ti] OR "Spatial Navigation"[majr] OR "spatial skills"[ti] OR "spatial skill"[ti] OR "visuospatial abilities"[ti] OR "visuospatial ability"[ti] OR "psychomotor abilities"[ti] OR "psychomotor ability"[ti] OR "motor abilities"[ti] OR "motor ability"[ti] OR "visuomotor abilities"[ti] OR "visuomotor ability"[ti] OR "spatial abilities"[ti] OR "spatial ability"[ti] OR "Task Performance and Analysis"[majr] OR "skills"[ti] OR "skill"[ti] OR "skilled"[ti]) AND ("training"[tw] OR "train*"[tw] OR "Learning"[mesh] OR "learning"[tw] OR "learn*"[tw] OR "Education"[mesh] OR "education"[subheading] OR "educat*"[tw] OR "Students"[mesh] OR "practice"[tw])) **OR** (("point of care ultrasonography"[tw] OR "point of care ultrasound"[tw] OR "point of care ultras*"[tw] OR "POCUS"[tw] OR (("Point-of-Care Testing"[Mesh] OR "Point-of-Care Systems"[Mesh] OR "point of care"[tw] OR "point of car*"[tw] OR "pointofcare"[tw]) AND ("Ultrasonography"[Mesh] OR "Ultrasonics"[mesh] OR "ultrasonography"[tw] OR "ultrasound"[tw] OR "ultras*"[tw] OR "sonography"[tw] OR "sonogra*"[tw] OR "echography"[tw] OR "echogra*"[tw])) OR "bedside ultrasonography"[tw] OR "bedside ultrasound"[tw] OR "bedside ultras*"[tw]) AND ("Space Perception"[Mesh] OR "Space Perception"[tw] OR "visuospatial skills"[tw] OR "visuospatial skill"[tw] OR "mental rotation"[tw] OR "perspective taking"[tw] OR "spatial perception"[tw] OR "Psychomotor Performance"[Mesh] OR "psychomotor skills"[tw] OR "psychomotor skill"[tw] OR "Motor Skills"[mesh] OR "motor skills"[tw] OR "motor skill"[tw] OR "dexterity"[tw] OR "visuomotor skills"[tw] OR "visuomotor skill"[tw] OR "Visual Perception"[mesh] OR "anatomical knowledge"[tw] OR "anatomy knowledge"[tw] OR "physiological knowledge"[tw] OR "physiology knowledge"[tw] OR "Spatial Navigation"[mesh] OR "spatial skills"[tw] OR "spatial skill"[tw] OR "visuospatial abilities"[tw] OR "visuospatial ability"[tw] OR "psychomotor abilities"[tw] OR "psychomotor ability"[tw] OR "motor abilities"[tw] OR "motor ability"[tw] OR "visuomotor abilities"[tw] OR "visuomotor ability"[tw] OR "spatial abilities"[tw] OR "spatial ability"[tw] OR "Task Performance and Analysis"[mesh] OR "skills"[tw] OR "skill"[tw] OR "skilled"[tw]) AND ("training"[tw] OR "train*"[tw] OR "Learning"[mesh] OR "learning"[tw] OR "learn*"[tw] OR "Education"[mesh] OR "education"[subheading] OR "educat*"[tw] OR "Students"[mesh] OR "practice"[tw]))**)** NOT ("Animals"[mesh] NOT "Humans"[mesh]) AND (english[la] OR dutch[la])

**Embase**

**(**((exp *"Echography"/ OR exp *"Ultrasound"/ OR "ultrasonography".ti OR "ultrasound".ti OR "ultras*".ti OR "sonography".ti OR "sonogra*".ti OR "echography".ti OR "echogra*".ti OR "echo".ti) AND (exp *"Depth Perception"/ OR "Space Perception".ti,ab OR "visuospatial skills".ti,ab OR "visuospatial skill".ti,ab OR "mental rotation".ti,ab OR "perspective taking".ti,ab OR "spatial perception".ti,ab OR exp *"Psychomotor Performance"/ OR "psychomotor skills".ti,ab OR "psychomotor skill".ti,ab OR exp *"Motor Performance"/ OR "motor skills".ti,ab OR "motor skill".ti,ab OR "dexterity".ti,ab OR "visuomotor skills".ti,ab OR "visuomotor skill".ti,ab OR exp *"Vision"/ OR "anatomical knowledge".ti,ab OR "anatomy knowledge".ti,ab OR "physiological knowledge".ti,ab OR "physiology knowledge".ti,ab OR exp *"Spatial orientation"/ OR "spatial skills".ti,ab OR "spatial skill".ti,ab OR "visuospatial abilities".ti,ab OR "visuospatial ability".ti,ab OR "psychomotor abilities".ti,ab OR "psychomotor ability".ti,ab OR "motor abilities".ti,ab OR "motor ability".ti,ab OR "visuomotor abilities".ti,ab OR "visuomotor ability".ti,ab OR "spatial abilities".ti,ab OR "spatial ability".ti,ab OR exp *"Task Performance"/ OR "skills".ti,ab OR "skill".ti,ab OR "skilled".ti,ab) AND ("training".ti,ab OR "train*".ti,ab OR exp *"Learning"/ OR "learning".ti,ab OR "learn*".ti,ab OR exp *"Education"/ OR "educat*".ti,ab OR exp *"Student"/ OR "practice".ti,ab)) **OR** ((exp *"Echography"/ OR exp *"Ultrasound"/ OR "ultrasonography".ti,ab OR "ultrasound".ti,ab OR "ultras*".ti,ab OR "sonography".ti,ab OR "sonogra*".ti,ab OR "echography".ti,ab OR "echogra*".ti,ab OR "echo".ti,ab) AND (exp *"Depth Perception"/ OR "Space Perception".ti OR "visuospatial skills".ti OR "visuospatial skill".ti OR "mental rotation".ti OR "perspective taking".ti OR "spatial perception".ti OR exp *"Psychomotor Performance"/ OR "psychomotor skills".ti OR "psychomotor skill".ti OR exp *"Motor Performance"/ OR "motor skills".ti OR "motor skill".ti OR "dexterity".ti OR "visuomotor skills".ti OR "visuomotor skill".ti OR exp *"Vision"/ OR "anatomical knowledge".ti OR "anatomy knowledge".ti OR "physiological knowledge".ti OR "physiology knowledge".ti OR exp *"Spatial orientation"/ OR "spatial skills".ti OR "spatial skill".ti OR "visuospatial abilities".ti OR "visuospatial ability".ti OR "psychomotor abilities".ti OR "psychomotor ability".ti OR "motor abilities".ti OR "motor ability".ti OR "visuomotor abilities".ti OR "visuomotor ability".ti OR "spatial abilities".ti OR "spatial ability".ti OR exp *"Task Performance"/ OR "skills".ti OR "skill".ti OR "skilled".ti) AND ("training".ti,ab OR "train*".ti,ab OR exp *"Learning"/ OR "learning".ti,ab OR "learn*".ti,ab OR exp *"Education"/ OR "educat*".ti,ab OR exp *"Student"/ OR "practice".ti,ab)) **OR** ((*"point of care ultrasound"/ OR "point of care ultrasonography".ti,ab OR "point of care ultrasound".ti,ab OR "point of care ultras*".ti,ab OR "POCUS".ti,ab OR ((*"Point of Care Testing"/ OR "Point of Care System"/ OR "point of care".ti,ab OR "point of car*".ti,ab OR "pointofcare".ti,ab) AND (exp *"Ultrasonography"/ OR exp *"Ultrasound"/ OR "ultrasonography".ti,ab OR "ultrasound".ti,ab OR "ultras*".ti,ab OR "sonography".ti,ab OR "sonogra*".ti,ab OR "echography".ti,ab OR "echogra*".ti,ab)) OR "bedside ultrasonography".ti,ab OR "bedside ultrasound".ti,ab OR "bedside ultras*".ti,ab) AND (exp *"Depth Perception"/ OR "Space Perception".ti,ab OR "visuospatial skills".ti,ab OR "visuospatial skill".ti,ab OR "mental rotation".ti,ab OR "perspective taking".ti,ab OR "spatial perception".ti,ab OR exp *"Psychomotor Performance"/ OR "psychomotor skills".ti,ab OR "psychomotor skill".ti,ab OR exp *"Motor performance"/ OR "motor skills".ti,ab OR "motor skill".ti,ab OR "dexterity".ti,ab OR "visuomotor skills".ti,ab OR "visuomotor skill".ti,ab OR exp *"Vision"/ OR "anatomical knowledge".ti,ab OR "anatomy knowledge".ti,ab OR "physiological knowledge".ti,ab OR "physiology knowledge".ti,ab OR exp *"Spatial orientation"/ OR "spatial skills".ti,ab OR "spatial skill".ti,ab OR "visuospatial abilities".ti,ab OR "visuospatial ability".ti,ab OR "psychomotor abilities".ti,ab OR "psychomotor ability".ti,ab OR "motor abilities".ti,ab OR "motor ability".ti,ab OR "visuomotor abilities".ti,ab OR "visuomotor ability".ti,ab OR "spatial abilities".ti,ab OR "spatial ability".ti,ab OR exp *"Task Performance"/ OR "skills".ti,ab OR "skill".ti,ab OR "skilled".ti,ab) AND ("training".ti,ab OR "train*".ti,ab OR exp *"Learning"/ OR "learning".ti,ab OR "learn*".ti,ab OR exp *"Education"/ OR "educat*".ti,ab OR exp "Student"/ OR "practice".ti,ab))**)** NOT (exp "Animals"/ NOT exp "Humans"/) AND (english.la OR dutch.la)

O NOT conference review.pt

o NOT (conference review or conference abstract).pt

o AND (conference abstract).pt

**Web of Science**

**((**(ti=("Echography" OR "Ultrasound" OR "ultrasonography" OR "ultrasound" OR "ultras*" OR "sonography" OR "sonogra*" OR "echography" OR "echogra*" OR "echo") AND ab=("Depth Perception" OR "Space Perception" OR "visuospatial skills" OR "visuospatial skill" OR "mental rotation" OR "perspective taking" OR "spatial perception" OR "Psychomotor Performance" OR "psychomotor skills" OR "psychomotor skill" OR "Motor Performance" OR "motor skills" OR "motor skill" OR "dexterity" OR "visuomotor skills" OR "visuomotor skill" OR "Vision" OR "anatomical knowledge" OR "anatomy knowledge" OR "physiological knowledge" OR "physiology knowledge" OR "Spatial orientation" OR "spatial skills" OR "spatial skill" OR "visuospatial abilities" OR "visuospatial ability" OR "psychomotor abilities" OR "psychomotor ability" OR "motor abilities" OR "motor ability" OR "visuomotor abilities" OR "visuomotor ability" OR "spatial abilities" OR "spatial ability" OR "Task Performance" OR "skills" OR "skill" OR "skilled") AND ab=("training" OR "train*" OR "Learning" OR "learning" OR "learn*" OR "Education" OR "educat*" OR "Student" OR "practice")) **OR** (ab=("Echography" OR "Ultrasound" OR "ultrasonography" OR "ultrasound" OR "ultras*" OR "sonography" OR "sonogra*" OR "echography" OR "echogra*" OR "echo") AND ti=("Depth Perception" OR "Space Perception" OR "visuospatial skills" OR "visuospatial skill" OR "mental rotation" OR "perspective taking" OR "spatial perception" OR "Psychomotor Performance" OR "psychomotor skills" OR "psychomotor skill" OR "Motor Performance" OR "motor skills" OR "motor skill" OR "dexterity" OR "visuomotor skills" OR "visuomotor skill" OR "Vision" OR "anatomical knowledge" OR "anatomy knowledge" OR "physiological knowledge" OR "physiology knowledge" OR "Spatial orientation" OR "spatial skills" OR "spatial skill" OR "visuospatial abilities" OR "visuospatial ability" OR "psychomotor abilities" OR "psychomotor ability" OR "motor abilities" OR "motor ability" OR "visuomotor abilities" OR "visuomotor ability" OR "spatial abilities" OR "spatial ability" OR "Task Performance" OR "skills" OR "skill" OR "skilled") AND ab=("training" OR "train*" OR "Learning" OR "learning" OR "learn*" OR "Education" OR "educat*" OR "Student" OR "practice")) **OR** ab=(("point of care ultrasonography" OR "point of care ultrasound" OR "point of care ultras*" OR "POCUS" OR (("Point-of-Care Testing" OR "Point-of-Care Systems" OR "point of care" OR "point of car*" OR "pointofcare") AND ("Ultrasonography" OR "Ultrasonics" OR "ultrasonography" OR "ultrasound" OR "ultras*" OR "sonography" OR "sonogra*" OR "echography" OR "echogra*")) OR "bedside ultrasonography" OR "bedside ultrasound" OR "bedside ultras*") AND ("Depth Perception" OR "Space Perception" OR "visuospatial skills" OR "visuospatial skill" OR "mental rotation" OR "perspective taking" OR "spatial perception" OR "Psychomotor Performance" OR "psychomotor skills" OR "psychomotor skill" OR "Motor performance" OR "motor skills" OR "motor skill" OR "dexterity" OR "visuomotor skills" OR "visuomotor skill" OR "Vision" OR "anatomical knowledge" OR "anatomy knowledge" OR "physiological knowledge" OR "physiology knowledge" OR "Spatial orientation" OR "spatial skills" OR "spatial skill" OR "visuospatial abilities" OR "visuospatial ability" OR "psychomotor abilities" OR "psychomotor ability" OR "motor abilities" OR "motor ability" OR "visuomotor abilities" OR "visuomotor ability" OR "spatial abilities" OR "spatial ability" OR "Task Performance" OR "skills" OR "skill" OR "skilled") AND ("training" OR "train*" OR "Learning" OR "learning" OR "learn*" OR "Education" OR "educat*" OR "Student" OR "practice"))**)** NOT ti=("veterinary" OR "rabbit" OR "rabbits" OR "animal" OR "animals" OR "mouse" OR "mice" OR "rodent" OR "rodents" OR "rat" OR "rats" OR "pig" OR "pigs" OR "porcine" OR "horse" OR "horses" OR "equine" OR "cow" OR "cows" OR "bovine" OR "goat" OR "goats" OR "sheep" OR "ovine" OR "canine" OR "dog" OR "dogs" OR "feline" OR "cat" OR "cats") AND la=(english OR dutch)**) OR ((**(ti=("Echography" OR "Ultrasound" OR "ultrasonography" OR "ultrasound" OR "ultras*" OR "sonography" OR "sonogra*" OR "echography" OR "echogra*" OR "echo") AND ti=("Depth Perception" OR "Space Perception" OR "visuospatial skills" OR "visuospatial skill" OR "mental rotation" OR "perspective taking" OR "spatial perception" OR "Psychomotor Performance" OR "psychomotor skills" OR "psychomotor skill" OR "Motor Performance" OR "motor skills" OR "motor skill" OR "dexterity" OR "visuomotor skills" OR "visuomotor skill" OR "Vision" OR "anatomical knowledge" OR "anatomy knowledge" OR "physiological knowledge" OR "physiology knowledge" OR "Spatial orientation" OR "spatial skills" OR "spatial skill" OR "visuospatial abilities" OR "visuospatial ability" OR "psychomotor abilities" OR "psychomotor ability" OR "motor abilities" OR "motor ability" OR "visuomotor abilities" OR "visuomotor ability" OR "spatial abilities" OR "spatial ability" OR "Task Performance" OR "skills" OR "skill" OR "skilled") AND ti=("training" OR "train*" OR "Learning" OR "learning" OR "learn*" OR "Education" OR "educat*" OR "Student" OR "practice")) **OR** (ti=("Echography" OR "Ultrasound" OR "ultrasonography" OR "ultrasound" OR "ultras*" OR "sonography" OR "sonogra*" OR "echography" OR "echogra*" OR "echo") AND ti=("Depth Perception" OR "Space Perception" OR "visuospatial skills" OR "visuospatial skill" OR "mental rotation" OR "perspective taking" OR "spatial perception" OR "Psychomotor Performance" OR "psychomotor skills" OR "psychomotor skill" OR "Motor Performance" OR "motor skills" OR "motor skill" OR "dexterity" OR "visuomotor skills" OR "visuomotor skill" OR "Vision" OR "anatomical knowledge" OR "anatomy knowledge" OR "physiological knowledge" OR "physiology knowledge" OR "Spatial orientation" OR "spatial skills" OR "spatial skill" OR "visuospatial abilities" OR "visuospatial ability" OR "psychomotor abilities" OR "psychomotor ability" OR "motor abilities" OR "motor ability" OR "visuomotor abilities" OR "visuomotor ability" OR "spatial abilities" OR "spatial ability" OR "Task Performance" OR "skills" OR "skill" OR "skilled") AND ti=("training" OR "train*" OR "Learning" OR "learning" OR "learn*" OR "Education" OR "educat*" OR "Student" OR "practice")) **OR** ti=(("point of care ultrasonography" OR "point of care ultrasound" OR "point of care ultras*" OR "POCUS" OR (("Point-of-Care Testing" OR "Point-of-Care Systems" OR "point of care" OR "point of car*" OR "pointofcare") AND ("Ultrasonography" OR "Ultrasonics" OR "ultrasonography" OR "ultrasound" OR "ultras*" OR "sonography" OR "sonogra*" OR "echography" OR "echogra*")) OR "bedside ultrasonography" OR "bedside ultrasound" OR "bedside ultras*") AND ("Depth Perception" OR "Space Perception" OR "visuospatial skills" OR "visuospatial skill" OR "mental rotation" OR "perspective taking" OR "spatial perception" OR "Psychomotor Performance" OR "psychomotor skills" OR "psychomotor skill" OR "Motor performance" OR "motor skills" OR "motor skill" OR "dexterity" OR "visuomotor skills" OR "visuomotor skill" OR "Vision" OR "anatomical knowledge" OR "anatomy knowledge" OR "physiological knowledge" OR "physiology knowledge" OR "Spatial orientation" OR "spatial skills" OR "spatial skill" OR "visuospatial abilities" OR "visuospatial ability" OR "psychomotor abilities" OR "psychomotor ability" OR "motor abilities" OR "motor ability" OR "visuomotor abilities" OR "visuomotor ability" OR "spatial abilities" OR "spatial ability" OR "Task Performance" OR "skills" OR "skill" OR "skilled") AND ("training" OR "train*" OR "Learning" OR "learning" OR "learn*" OR "Education" OR "educat*" OR "Student" OR "practice"))**)** NOT ti=("veterinary" OR "rabbit" OR "rabbits" OR "animal" OR "animals" OR "mouse" OR "mice" OR "rodent" OR "rodents" OR "rat" OR "rats" OR "pig" OR "pigs" OR "porcine" OR "horse" OR "horses" OR "equine" OR "cow" OR "cows" OR "bovine" OR "goat" OR "goats" OR "sheep" OR "ovine" OR "canine" OR "dog" OR "dogs" OR "feline" OR "cat" OR "cats") AND la=(english OR dutch)**)**

**Cochrane**

**(**(("Echography" OR "Ultrasound" OR "ultrasonography" OR "ultrasound" OR "ultras*" OR "sonography" OR "sonogra*" OR "echography" OR "echogra*" OR "echo"):ti AND ("Depth Perception" OR "Space Perception" OR "visuospatial skills" OR "visuospatial skill" OR "mental rotation" OR "perspective taking" OR "spatial perception" OR "Psychomotor Performance" OR "psychomotor skills" OR "psychomotor skill" OR "Motor Performance" OR "motor skills" OR "motor skill" OR "dexterity" OR "visuomotor skills" OR "visuomotor skill" OR "Vision" OR "anatomical knowledge" OR "anatomy knowledge" OR "physiological knowledge" OR "physiology knowledge" OR "Spatial orientation" OR "spatial skills" OR "spatial skill" OR "visuospatial abilities" OR "visuospatial ability" OR "psychomotor abilities" OR "psychomotor ability" OR "motor abilities" OR "motor ability" OR "visuomotor abilities" OR "visuomotor ability" OR "spatial abilities" OR "spatial ability" OR "Task Performance" OR "skills" OR "skill" OR "skilled"):ti,ab,kw AND ("training" OR "train*" OR "Learning" OR "learning" OR "learn*" OR "Education" OR "educat*" OR "Student" OR "practice"):ti,ab,kw) **OR** (("Echography" OR "Ultrasound" OR "ultrasonography" OR "ultrasound" OR "ultras*" OR "sonography" OR "sonogra*" OR "echography" OR "echogra*" OR "echo"):ti,ab,kw AND ("Depth Perception" OR "Space Perception" OR "visuospatial skills" OR "visuospatial skill" OR "mental rotation" OR "perspective taking" OR "spatial perception" OR "Psychomotor Performance" OR "psychomotor skills" OR "psychomotor skill" OR "Motor Performance" OR "motor skills" OR "motor skill" OR "dexterity" OR "visuomotor skills" OR "visuomotor skill" OR "Vision" OR "anatomical knowledge" OR "anatomy knowledge" OR "physiological knowledge" OR "physiology knowledge" OR "Spatial orientation" OR "spatial skills" OR "spatial skill" OR "visuospatial abilities" OR "visuospatial ability" OR "psychomotor abilities" OR "psychomotor ability" OR "motor abilities" OR "motor ability" OR "visuomotor abilities" OR "visuomotor ability" OR "spatial abilities" OR "spatial ability" OR "Task Performance" OR "skills" OR "skill" OR "skilled"):ti AND ("training" OR "train*" OR "Learning" OR "learning" OR "learn*" OR "Education" OR "educat*" OR "Student" OR "practice"):ti,ab,kw) **OR** (("point of care ultrasonography" OR "point of care ultrasound" OR "point of care ultras*" OR "POCUS" OR (("Point of Care Testing" OR "Point of Care Systems" OR "point of care" OR "point of car*" OR "pointofcare") AND ("Ultrasonography" OR "Ultrasonics" OR "ultrasonography" OR "ultrasound" OR "ultras*" OR "sonography" OR "sonogra*" OR "echography" OR "echogra*")) OR "bedside ultrasonography" OR "bedside ultrasound" OR "bedside ultras*") AND ("Depth Perception" OR "Space Perception" OR "visuospatial skills" OR "visuospatial skill" OR "mental rotation" OR "perspective taking" OR "spatial perception" OR "Psychomotor Performance" OR "psychomotor skills" OR "psychomotor skill" OR "Motor performance" OR "motor skills" OR "motor skill" OR "dexterity" OR "visuomotor skills" OR "visuomotor skill" OR "Vision" OR "anatomical knowledge" OR "anatomy knowledge" OR "physiological knowledge" OR "physiology knowledge" OR "Spatial orientation" OR "spatial skills" OR "spatial skill" OR "visuospatial abilities" OR "visuospatial ability" OR "psychomotor abilities" OR "psychomotor ability" OR "motor abilities" OR "motor ability" OR "visuomotor abilities" OR "visuomotor ability" OR "spatial abilities" OR "spatial ability" OR "Task Performance" OR "skills" OR "skill" OR "skilled") AND ("training" OR "train*" OR "Learning" OR "learning" OR "learn*" OR "Education" OR "educat*" OR "Student" OR "practice")):ti,ab,kw**)**

**Emcare**

**(**((exp *"Echography"/ OR exp *"Ultrasound"/ OR "ultrasonography".ti OR "ultrasound".ti OR "ultras*".ti OR "sonography".ti OR "sonogra*".ti OR "echography".ti OR "echogra*".ti OR "echo".ti) AND (exp *"Depth Perception"/ OR "Space Perception".ti,ab OR "visuospatial skills".ti,ab OR "visuospatial skill".ti,ab OR "mental rotation".ti,ab OR "perspective taking".ti,ab OR "spatial perception".ti,ab OR exp *"Psychomotor Performance"/ OR "psychomotor skills".ti,ab OR "psychomotor skill".ti,ab OR exp *"Motor Performance"/ OR "motor skills".ti,ab OR "motor skill".ti,ab OR "dexterity".ti,ab OR "visuomotor skills".ti,ab OR "visuomotor skill".ti,ab OR exp *"Vision"/ OR "anatomical knowledge".ti,ab OR "anatomy knowledge".ti,ab OR "physiological knowledge".ti,ab OR "physiology knowledge".ti,ab OR exp *"Spatial orientation"/ OR "spatial skills".ti,ab OR "spatial skill".ti,ab OR "visuospatial abilities".ti,ab OR "visuospatial ability".ti,ab OR "psychomotor abilities".ti,ab OR "psychomotor ability".ti,ab OR "motor abilities".ti,ab OR "motor ability".ti,ab OR "visuomotor abilities".ti,ab OR "visuomotor ability".ti,ab OR "spatial abilities".ti,ab OR "spatial ability".ti,ab OR exp *"Task Performance"/ OR "skills".ti,ab OR "skill".ti,ab OR "skilled".ti,ab) AND ("training".ti,ab OR "train*".ti,ab OR exp *"Learning"/ OR "learning".ti,ab OR "learn*".ti,ab OR exp *"Education"/ OR "educat*".ti,ab OR exp *"Student"/ OR "practice".ti,ab)) **OR** ((exp *"Echography"/ OR exp *"Ultrasound"/ OR "ultrasonography".ti,ab OR "ultrasound".ti,ab OR "ultras*".ti,ab OR "sonography".ti,ab OR "sonogra*".ti,ab OR "echography".ti,ab OR "echogra*".ti,ab OR "echo".ti,ab) AND (exp *"Depth Perception"/ OR "Space Perception".ti OR "visuospatial skills".ti OR "visuospatial skill".ti OR "mental rotation".ti OR "perspective taking".ti OR "spatial perception".ti OR exp *"Psychomotor Performance"/ OR "psychomotor skills".ti OR "psychomotor skill".ti OR exp *"Motor Performance"/ OR "motor skills".ti OR "motor skill".ti OR "dexterity".ti OR "visuomotor skills".ti OR "visuomotor skill".ti OR exp *"Vision"/ OR "anatomical knowledge".ti OR "anatomy knowledge".ti OR "physiological knowledge".ti OR "physiology knowledge".ti OR exp *"Spatial orientation"/ OR "spatial skills".ti OR "spatial skill".ti OR "visuospatial abilities".ti OR "visuospatial ability".ti OR "psychomotor abilities".ti OR "psychomotor ability".ti OR "motor abilities".ti OR "motor ability".ti OR "visuomotor abilities".ti OR "visuomotor ability".ti OR "spatial abilities".ti OR "spatial ability".ti OR exp *"Task Performance"/ OR "skills".ti OR "skill".ti OR "skilled".ti) AND ("training".ti,ab OR "train*".ti,ab OR exp *"Learning"/ OR "learning".ti,ab OR "learn*".ti,ab OR exp *"Education"/ OR "educat*".ti,ab OR exp *"Student"/ OR "practice".ti,ab)) **OR** (("point of care ultrasonography".ti,ab OR "point of care ultrasound".ti,ab OR "point of care ultras*".ti,ab OR "POCUS".ti,ab OR (("Point-of-Care Testing"/ OR "Point-of-Care Systems"/ OR "point of care".ti,ab OR "point of car*".ti,ab OR "pointofcare".ti,ab) AND ("Ultrasonography"/ OR "Ultrasonics"/ OR "ultrasonography".ti,ab OR "ultrasound".ti,ab OR "ultras*".ti,ab OR "sonography".ti,ab OR "sonogra*".ti,ab OR "echography".ti,ab OR "echogra*".ti,ab)) OR "bedside ultrasonography".ti,ab OR "bedside ultrasound".ti,ab OR "bedside ultras*".ti,ab) AND (exp *"Depth Perception"/ OR "Space Perception".ti,ab OR "visuospatial skills".ti,ab OR "visuospatial skill".ti,ab OR "mental rotation".ti,ab OR "perspective taking".ti,ab OR "spatial perception".ti,ab OR exp *"Psychomotor Performance"/ OR "psychomotor skills".ti,ab OR "psychomotor skill".ti,ab OR exp *"Motor performance"/ OR "motor skills".ti,ab OR "motor skill".ti,ab OR "dexterity".ti,ab OR "visuomotor skills".ti,ab OR "visuomotor skill".ti,ab OR exp *"Vision"/ OR "anatomical knowledge".ti,ab OR "anatomy knowledge".ti,ab OR "physiological knowledge".ti,ab OR "physiology knowledge".ti,ab OR exp *"Spatial orientation"/ OR "spatial skills".ti,ab OR "spatial skill".ti,ab OR "visuospatial abilities".ti,ab OR "visuospatial ability".ti,ab OR "psychomotor abilities".ti,ab OR "psychomotor ability".ti,ab OR "motor abilities".ti,ab OR "motor ability".ti,ab OR "visuomotor abilities".ti,ab OR "visuomotor ability".ti,ab OR "spatial abilities".ti,ab OR "spatial ability".ti,ab OR exp *"Task Performance"/ OR "skills".ti,ab OR "skill".ti,ab OR "skilled".ti,ab) AND ("training".ti,ab OR "train*".ti,ab OR exp *"Learning"/ OR "learning".ti,ab OR "learn*".ti,ab OR exp *"Education"/ OR "educat*".ti,ab OR exp "Student"/ OR "practice".ti,ab))**)** NOT (exp "Animals"/ NOT exp "Humans"/) AND (english.la OR dutch.la)

**PsycINFO**

**((**(TI("Echography" OR "Ultrasound" OR "ultrasonography" OR "ultrasound" OR "ultras*" OR "sonography" OR "sonogra*" OR "echography" OR "echogra*" OR "echo") AND TX("Depth Perception" OR "Space Perception" OR "visuospatial skills" OR "visuospatial skill" OR "mental rotation" OR "perspective taking" OR "spatial perception" OR "Psychomotor Performance" OR "psychomotor skills" OR "psychomotor skill" OR "Motor Performance" OR "motor skills" OR "motor skill" OR "dexterity" OR "visuomotor skills" OR "visuomotor skill" OR "Vision" OR "anatomical knowledge" OR "anatomy knowledge" OR "physiological knowledge" OR "physiology knowledge" OR "Spatial orientation" OR "spatial skills" OR "spatial skill" OR "visuospatial abilities" OR "visuospatial ability" OR "psychomotor abilities" OR "psychomotor ability" OR "motor abilities" OR "motor ability" OR "visuomotor abilities" OR "visuomotor ability" OR "spatial abilities" OR "spatial ability" OR "Task Performance" OR "skills" OR "skill" OR "skilled") AND TX("training" OR "train*" OR "Learning" OR "learning" OR "learn*" OR "Education" OR "educat*" OR "Student" OR "practice")) **OR** (TX("Echography" OR "Ultrasound" OR "ultrasonography" OR "ultrasound" OR "ultras*" OR "sonography" OR "sonogra*" OR "echography" OR "echogra*" OR "echo") AND TI("Depth Perception" OR "Space Perception" OR "visuospatial skills" OR "visuospatial skill" OR "mental rotation" OR "perspective taking" OR "spatial perception" OR "Psychomotor Performance" OR "psychomotor skills" OR "psychomotor skill" OR "Motor Performance" OR "motor skills" OR "motor skill" OR "dexterity" OR "visuomotor skills" OR "visuomotor skill" OR "Vision" OR "anatomical knowledge" OR "anatomy knowledge" OR "physiological knowledge" OR "physiology knowledge" OR "Spatial orientation" OR "spatial skills" OR "spatial skill" OR "visuospatial abilities" OR "visuospatial ability" OR "psychomotor abilities" OR "psychomotor ability" OR "motor abilities" OR "motor ability" OR "visuomotor abilities" OR "visuomotor ability" OR "spatial abilities" OR "spatial ability" OR "Task Performance" OR "skills" OR "skill" OR "skilled") AND TX("training" OR "train*" OR "Learning" OR "learning" OR "learn*" OR "Education" OR "educat*" OR "Student" OR "practice")) **OR** TX(("point of care ultrasonography" OR "point of care ultrasound" OR "point of care ultras*" OR "POCUS" OR (("Point-of-Care Testing" OR "Point-of-Care Systems" OR "point of care" OR "point of car*" OR "pointofcare") AND ("Ultrasonography" OR "Ultrasonics" OR "ultrasonography" OR "ultrasound" OR "ultras*" OR "sonography" OR "sonogra*" OR "echography" OR "echogra*")) OR "bedside ultrasonography" OR "bedside ultrasound" OR "bedside ultras*") AND ("Depth Perception" OR "Space Perception" OR "visuospatial skills" OR "visuospatial skill" OR "mental rotation" OR "perspective taking" OR "spatial perception" OR "Psychomotor Performance" OR "psychomotor skills" OR "psychomotor skill" OR "Motor performance" OR "motor skills" OR "motor skill" OR "dexterity" OR "visuomotor skills" OR "visuomotor skill" OR "Vision" OR "anatomical knowledge" OR "anatomy knowledge" OR "physiological knowledge" OR "physiology knowledge" OR "Spatial orientation" OR "spatial skills" OR "spatial skill" OR "visuospatial abilities" OR "visuospatial ability" OR "psychomotor abilities" OR "psychomotor ability" OR "motor abilities" OR "motor ability" OR "visuomotor abilities" OR "visuomotor ability" OR "spatial abilities" OR "spatial ability" OR "Task Performance" OR "skills" OR "skill" OR "skilled") AND ("training" OR "train*" OR "Learning" OR "learning" OR "learn*" OR "Education" OR "educat*" OR "Student" OR "practice"))**)** NOT TI("veterinary" OR "rabbit" OR "rabbits" OR "animal" OR "animals" OR "mouse" OR "mice" OR "rodent" OR "rodents" OR "rat" OR "rats" OR "pig" OR "pigs" OR "porcine" OR "horse" OR "horses" OR "equine" OR "cow" OR "cows" OR "bovine" OR "goat" OR "goats" OR "sheep" OR "ovine" OR "canine" OR "dog" OR "dogs" OR "feline" OR "cat" OR "cats") AND la=(english OR dutch)**) OR ((**(TI("Echography" OR "Ultrasound" OR "ultrasonography" OR "ultrasound" OR "ultras*" OR "sonography" OR "sonogra*" OR "echography" OR "echogra*" OR "echo") AND TI("Depth Perception" OR "Space Perception" OR "visuospatial skills" OR "visuospatial skill" OR "mental rotation" OR "perspective taking" OR "spatial perception" OR "Psychomotor Performance" OR "psychomotor skills" OR "psychomotor skill" OR "Motor Performance" OR "motor skills" OR "motor skill" OR "dexterity" OR "visuomotor skills" OR "visuomotor skill" OR "Vision" OR "anatomical knowledge" OR "anatomy knowledge" OR "physiological knowledge" OR "physiology knowledge" OR "Spatial orientation" OR "spatial skills" OR "spatial skill" OR "visuospatial abilities" OR "visuospatial ability" OR "psychomotor abilities" OR "psychomotor ability" OR "motor abilities" OR "motor ability" OR "visuomotor abilities" OR "visuomotor ability" OR "spatial abilities" OR "spatial ability" OR "Task Performance" OR "skills" OR "skill" OR "skilled") AND TI("training" OR "train*" OR "Learning" OR "learning" OR "learn*" OR "Education" OR "educat*" OR "Student" OR "practice")) **OR** (TI("Echography" OR "Ultrasound" OR "ultrasonography" OR "ultrasound" OR "ultras*" OR "sonography" OR "sonogra*" OR "echography" OR "echogra*" OR "echo") AND TI("Depth Perception" OR "Space Perception" OR "visuospatial skills" OR "visuospatial skill" OR "mental rotation" OR "perspective taking" OR "spatial perception" OR "Psychomotor Performance" OR "psychomotor skills" OR "psychomotor skill" OR "Motor Performance" OR "motor skills" OR "motor skill" OR "dexterity" OR "visuomotor skills" OR "visuomotor skill" OR "Vision" OR "anatomical knowledge" OR "anatomy knowledge" OR "physiological knowledge" OR "physiology knowledge" OR "Spatial orientation" OR "spatial skills" OR "spatial skill" OR "visuospatial abilities" OR "visuospatial ability" OR "psychomotor abilities" OR "psychomotor ability" OR "motor abilities" OR "motor ability" OR "visuomotor abilities" OR "visuomotor ability" OR "spatial abilities" OR "spatial ability" OR "Task Performance" OR "skills" OR "skill" OR "skilled") AND TI("training" OR "train*" OR "Learning" OR "learning" OR "learn*" OR "Education" OR "educat*" OR "Student" OR "practice")) **OR** TI(("point of care ultrasonography" OR "point of care ultrasound" OR "point of care ultras*" OR "POCUS" OR (("Point-of-Care Testing" OR "Point-of-Care Systems" OR "point of care" OR "point of car*" OR "pointofcare") AND ("Ultrasonography" OR "Ultrasonics" OR "ultrasonography" OR "ultrasound" OR "ultras*" OR "sonography" OR "sonogra*" OR "echography" OR "echogra*")) OR "bedside ultrasonography" OR "bedside ultrasound" OR "bedside ultras*") AND ("Depth Perception" OR "Space Perception" OR "visuospatial skills" OR "visuospatial skill" OR "mental rotation" OR "perspective taking" OR "spatial perception" OR "Psychomotor Performance" OR "psychomotor skills" OR "psychomotor skill" OR "Motor performance" OR "motor skills" OR "motor skill" OR "dexterity" OR "visuomotor skills" OR "visuomotor skill" OR "Vision" OR "anatomical knowledge" OR "anatomy knowledge" OR "physiological knowledge" OR "physiology knowledge" OR "Spatial orientation" OR "spatial skills" OR "spatial skill" OR "visuospatial abilities" OR "visuospatial ability" OR "psychomotor abilities" OR "psychomotor ability" OR "motor abilities" OR "motor ability" OR "visuomotor abilities" OR "visuomotor ability" OR "spatial abilities" OR "spatial ability" OR "Task Performance" OR "skills" OR "skill" OR "skilled") AND ("training" OR "train*" OR "Learning" OR "learning" OR "learn*" OR "Education" OR "educat*" OR "Student" OR "practice"))**)** NOT TI("veterinary" OR "rabbit" OR "rabbits" OR "animal" OR "animals" OR "mouse" OR "mice" OR "rodent" OR "rodents" OR "rat" OR "rats" OR "pig" OR "pigs" OR "porcine" OR "horse" OR "horses" OR "equine" OR "cow" OR "cows" OR "bovine" OR "goat" OR "goats" OR "sheep" OR "ovine" OR "canine" OR "dog" OR "dogs" OR "feline" OR "cat" OR "cats")**)**

AND la=(english OR dutch

**Academic Search Premier**

**((**(TI("Echography" OR "Ultrasound" OR "ultrasonography" OR "ultrasound" OR "ultras*" OR "sonography" OR "sonogra*" OR "echography" OR "echogra*" OR "echo") AND TX("Depth Perception" OR "Space Perception" OR "visuospatial skills" OR "visuospatial skill" OR "mental rotation" OR "perspective taking" OR "spatial perception" OR "Psychomotor Performance" OR "psychomotor skills" OR "psychomotor skill" OR "Motor Performance" OR "motor skills" OR "motor skill" OR "dexterity" OR "visuomotor skills" OR "visuomotor skill" OR "Vision" OR "anatomical knowledge" OR "anatomy knowledge" OR "physiological knowledge" OR "physiology knowledge" OR "Spatial orientation" OR "spatial skills" OR "spatial skill" OR "visuospatial abilities" OR "visuospatial ability" OR "psychomotor abilities" OR "psychomotor ability" OR "motor abilities" OR "motor ability" OR "visuomotor abilities" OR "visuomotor ability" OR "spatial abilities" OR "spatial ability" OR "Task Performance" OR "skills" OR "skill" OR "skilled") AND TX("training" OR "train*" OR "Learning" OR "learning" OR "learn*" OR "Education" OR "educat*" OR "Student" OR "practice")) **OR** (TX("Echography" OR "Ultrasound" OR "ultrasonography" OR "ultrasound" OR "ultras*" OR "sonography" OR "sonogra*" OR "echography" OR "echogra*" OR "echo") AND TI("Depth Perception" OR "Space Perception" OR "visuospatial skills" OR "visuospatial skill" OR "mental rotation" OR "perspective taking" OR "spatial perception" OR "Psychomotor Performance" OR "psychomotor skills" OR "psychomotor skill" OR "Motor Performance" OR "motor skills" OR "motor skill" OR "dexterity" OR "visuomotor skills" OR "visuomotor skill" OR "Vision" OR "anatomical knowledge" OR "anatomy knowledge" OR "physiological knowledge" OR "physiology knowledge" OR "Spatial orientation" OR "spatial skills" OR "spatial skill" OR "visuospatial abilities" OR "visuospatial ability" OR "psychomotor abilities" OR "psychomotor ability" OR "motor abilities" OR "motor ability" OR "visuomotor abilities" OR "visuomotor ability" OR "spatial abilities" OR "spatial ability" OR "Task Performance" OR "skills" OR "skill" OR "skilled") AND TX("training" OR "train*" OR "Learning" OR "learning" OR "learn*" OR "Education" OR "educat*" OR "Student" OR "practice")) **OR** TX(("point of care ultrasonography" OR "point of care ultrasound" OR "point of care ultras*" OR "POCUS" OR (("Point-of-Care Testing" OR "Point-of-Care Systems" OR "point of care" OR "point of car*" OR "pointofcare") AND ("Ultrasonography" OR "Ultrasonics" OR "ultrasonography" OR "ultrasound" OR "ultras*" OR "sonography" OR "sonogra*" OR "echography" OR "echogra*")) OR "bedside ultrasonography" OR "bedside ultrasound" OR "bedside ultras*") AND ("Depth Perception" OR "Space Perception" OR "visuospatial skills" OR "visuospatial skill" OR "mental rotation" OR "perspective taking" OR "spatial perception" OR "Psychomotor Performance" OR "psychomotor skills" OR "psychomotor skill" OR "Motor performance" OR "motor skills" OR "motor skill" OR "dexterity" OR "visuomotor skills" OR "visuomotor skill" OR "Vision" OR "anatomical knowledge" OR "anatomy knowledge" OR "physiological knowledge" OR "physiology knowledge" OR "Spatial orientation" OR "spatial skills" OR "spatial skill" OR "visuospatial abilities" OR "visuospatial ability" OR "psychomotor abilities" OR "psychomotor ability" OR "motor abilities" OR "motor ability" OR "visuomotor abilities" OR "visuomotor ability" OR "spatial abilities" OR "spatial ability" OR "Task Performance" OR "skills" OR "skill" OR "skilled") AND ("training" OR "train*" OR "Learning" OR "learning" OR "learn*" OR "Education" OR "educat*" OR "Student" OR "practice"))**)** NOT TI("veterinary" OR "rabbit" OR "rabbits" OR "animal" OR "animals" OR "mouse" OR "mice" OR "rodent" OR "rodents" OR "rat" OR "rats" OR "pig" OR "pigs" OR "porcine" OR "horse" OR "horses" OR "equine" OR "cow" OR "cows" OR "bovine" OR "goat" OR "goats" OR "sheep" OR "ovine" OR "canine" OR "dog" OR "dogs" OR "feline" OR "cat" OR "cats") AND la=(english OR dutch)**) OR ((**(TI("Echography" OR "Ultrasound" OR "ultrasonography" OR "ultrasound" OR "ultras*" OR "sonography" OR "sonogra*" OR "echography" OR "echogra*" OR "echo") AND TI("Depth Perception" OR "Space Perception" OR "visuospatial skills" OR "visuospatial skill" OR "mental rotation" OR "perspective taking" OR "spatial perception" OR "Psychomotor Performance" OR "psychomotor skills" OR "psychomotor skill" OR "Motor Performance" OR "motor skills" OR "motor skill" OR "dexterity" OR "visuomotor skills" OR "visuomotor skill" OR "Vision" OR "anatomical knowledge" OR "anatomy knowledge" OR "physiological knowledge" OR "physiology knowledge" OR "Spatial orientation" OR "spatial skills" OR "spatial skill" OR "visuospatial abilities" OR "visuospatial ability" OR "psychomotor abilities" OR "psychomotor ability" OR "motor abilities" OR "motor ability" OR "visuomotor abilities" OR "visuomotor ability" OR "spatial abilities" OR "spatial ability" OR "Task Performance" OR "skills" OR "skill" OR "skilled") AND TI("training" OR "train*" OR "Learning" OR "learning" OR "learn*" OR "Education" OR "educat*" OR "Student" OR "practice")) **OR** (TI("Echography" OR "Ultrasound" OR "ultrasonography" OR "ultrasound" OR "ultras*" OR "sonography" OR "sonogra*" OR "echography" OR "echogra*" OR "echo") AND TI("Depth Perception" OR "Space Perception" OR "visuospatial skills" OR "visuospatial skill" OR "mental rotation" OR "perspective taking" OR "spatial perception" OR "Psychomotor Performance" OR "psychomotor skills" OR "psychomotor skill" OR "Motor Performance" OR "motor skills" OR "motor skill" OR "dexterity" OR "visuomotor skills" OR "visuomotor skill" OR "Vision" OR "anatomical knowledge" OR "anatomy knowledge" OR "physiological knowledge" OR "physiology knowledge" OR "Spatial orientation" OR "spatial skills" OR "spatial skill" OR "visuospatial abilities" OR "visuospatial ability" OR "psychomotor abilities" OR "psychomotor ability" OR "motor abilities" OR "motor ability" OR "visuomotor abilities" OR "visuomotor ability" OR "spatial abilities" OR "spatial ability" OR "Task Performance" OR "skills" OR "skill" OR "skilled") AND TI("training" OR "train*" OR "Learning" OR "learning" OR "learn*" OR "Education" OR "educat*" OR "Student" OR "practice")) **OR** TI(("point of care ultrasonography" OR "point of care ultrasound" OR "point of care ultras*" OR "POCUS" OR (("Point-of-Care Testing" OR "Point-of-Care Systems" OR "point of care" OR "point of car*" OR "pointofcare") AND ("Ultrasonography" OR "Ultrasonics" OR "ultrasonography" OR "ultrasound" OR "ultras*" OR "sonography" OR "sonogra*" OR "echography" OR "echogra*")) OR "bedside ultrasonography" OR "bedside ultrasound" OR "bedside ultras*") AND ("Depth Perception" OR "Space Perception" OR "visuospatial skills" OR "visuospatial skill" OR "mental rotation" OR "perspective taking" OR "spatial perception" OR "Psychomotor Performance" OR "psychomotor skills" OR "psychomotor skill" OR "Motor performance" OR "motor skills" OR "motor skill" OR "dexterity" OR "visuomotor skills" OR "visuomotor skill" OR "Vision" OR "anatomical knowledge" OR "anatomy knowledge" OR "physiological knowledge" OR "physiology knowledge" OR "Spatial orientation" OR "spatial skills" OR "spatial skill" OR "visuospatial abilities" OR "visuospatial ability" OR "psychomotor abilities" OR "psychomotor ability" OR "motor abilities" OR "motor ability" OR "visuomotor abilities" OR "visuomotor ability" OR "spatial abilities" OR "spatial ability" OR "Task Performance" OR "skills" OR "skill" OR "skilled") AND ("training" OR "train*" OR "Learning" OR "learning" OR "learn*" OR "Education" OR "educat*" OR "Student" OR "practice"))**)** NOT TI("veterinary" OR "rabbit" OR "rabbits" OR "animal" OR "animals" OR "mouse" OR "mice" OR "rodent" OR "rodents" OR "rat" OR "rats" OR "pig" OR "pigs" OR "porcine" OR "horse" OR "horses" OR "equine" OR "cow" OR "cows" OR "bovine" OR "goat" OR "goats" OR "sheep" OR "ovine" OR "canine" OR "dog" OR "dogs" OR "feline" OR "cat" OR "cats")**)**

**ERIC**

**((**(TI("Echography" OR "Ultrasound" OR "ultrasonography" OR "ultrasound" OR "ultras*" OR "sonography" OR "sonogra*" OR "echography" OR "echogra*" OR "echo") AND TX("Depth Perception" OR "Space Perception" OR "visuospatial skills" OR "visuospatial skill" OR "mental rotation" OR "perspective taking" OR "spatial perception" OR "Psychomotor Performance" OR "psychomotor skills" OR "psychomotor skill" OR "Motor Performance" OR "motor skills" OR "motor skill" OR "dexterity" OR "visuomotor skills" OR "visuomotor skill" OR "Vision" OR "anatomical knowledge" OR "anatomy knowledge" OR "physiological knowledge" OR "physiology knowledge" OR "Spatial orientation" OR "spatial skills" OR "spatial skill" OR "visuospatial abilities" OR "visuospatial ability" OR "psychomotor abilities" OR "psychomotor ability" OR "motor abilities" OR "motor ability" OR "visuomotor abilities" OR "visuomotor ability" OR "spatial abilities" OR "spatial ability" OR "Task Performance" OR "skills" OR "skill" OR "skilled") AND TX("training" OR "train*" OR "Learning" OR "learning" OR "learn*" OR "Education" OR "educat*" OR "Student" OR "practice")) **OR** (TX("Echography" OR "Ultrasound" OR "ultrasonography" OR "ultrasound" OR "ultras*" OR "sonography" OR "sonogra*" OR "echography" OR "echogra*" OR "echo") AND TI("Depth Perception" OR "Space Perception" OR "visuospatial skills" OR "visuospatial skill" OR "mental rotation" OR "perspective taking" OR "spatial perception" OR "Psychomotor Performance" OR "psychomotor skills" OR "psychomotor skill" OR "Motor Performance" OR "motor skills" OR "motor skill" OR "dexterity" OR "visuomotor skills" OR "visuomotor skill" OR "Vision" OR "anatomical knowledge" OR "anatomy knowledge" OR "physiological knowledge" OR "physiology knowledge" OR "Spatial orientation" OR "spatial skills" OR "spatial skill" OR "visuospatial abilities" OR "visuospatial ability" OR "psychomotor abilities" OR "psychomotor ability" OR "motor abilities" OR "motor ability" OR "visuomotor abilities" OR "visuomotor ability" OR "spatial abilities" OR "spatial ability" OR "Task Performance" OR "skills" OR "skill" OR "skilled") AND TX("training" OR "train*" OR "Learning" OR "learning" OR "learn*" OR "Education" OR "educat*" OR "Student" OR "practice")) **OR** TX(("point of care ultrasonography" OR "point of care ultrasound" OR "point of care ultras*" OR "POCUS" OR (("Point-of-Care Testing" OR "Point-of-Care Systems" OR "point of care" OR "point of car*" OR "pointofcare") AND ("Ultrasonography" OR "Ultrasonics" OR "ultrasonography" OR "ultrasound" OR "ultras*" OR "sonography" OR "sonogra*" OR "echography" OR "echogra*")) OR "bedside ultrasonography" OR "bedside ultrasound" OR "bedside ultras*") AND ("Depth Perception" OR "Space Perception" OR "visuospatial skills" OR "visuospatial skill" OR "mental rotation" OR "perspective taking" OR "spatial perception" OR "Psychomotor Performance" OR "psychomotor skills" OR "psychomotor skill" OR "Motor performance" OR "motor skills" OR "motor skill" OR "dexterity" OR "visuomotor skills" OR "visuomotor skill" OR "Vision" OR "anatomical knowledge" OR "anatomy knowledge" OR "physiological knowledge" OR "physiology knowledge" OR "Spatial orientation" OR "spatial skills" OR "spatial skill" OR "visuospatial abilities" OR "visuospatial ability" OR "psychomotor abilities" OR "psychomotor ability" OR "motor abilities" OR "motor ability" OR "visuomotor abilities" OR "visuomotor ability" OR "spatial abilities" OR "spatial ability" OR "Task Performance" OR "skills" OR "skill" OR "skilled") AND ("training" OR "train*" OR "Learning" OR "learning" OR "learn*" OR "Education" OR "educat*" OR "Student" OR "practice"))**)** NOT TI("veterinary" OR "rabbit" OR "rabbits" OR "animal" OR "animals" OR "mouse" OR "mice" OR "rodent" OR "rodents" OR "rat" OR "rats" OR "pig" OR "pigs" OR "porcine" OR "horse" OR "horses" OR "equine" OR "cow" OR "cows" OR "bovine" OR "goat" OR "goats" OR "sheep" OR "ovine" OR "canine" OR "dog" OR "dogs" OR "feline" OR "cat" OR "cats") AND la=(english OR dutch)**) OR ((**(TI("Echography" OR "Ultrasound" OR "ultrasonography" OR "ultrasound" OR "ultras*" OR "sonography" OR "sonogra*" OR "echography" OR "echogra*" OR "echo") AND TI("Depth Perception" OR "Space Perception" OR "visuospatial skills" OR "visuospatial skill" OR "mental rotation" OR "perspective taking" OR "spatial perception" OR "Psychomotor Performance" OR "psychomotor skills" OR "psychomotor skill" OR "Motor Performance" OR "motor skills" OR "motor skill" OR "dexterity" OR "visuomotor skills" OR "visuomotor skill" OR "Vision" OR "anatomical knowledge" OR "anatomy knowledge" OR "physiological knowledge" OR "physiology knowledge" OR "Spatial orientation" OR "spatial skills" OR "spatial skill" OR "visuospatial abilities" OR "visuospatial ability" OR "psychomotor abilities" OR "psychomotor ability" OR "motor abilities" OR "motor ability" OR "visuomotor abilities" OR "visuomotor ability" OR "spatial abilities" OR "spatial ability" OR "Task Performance" OR "skills" OR "skill" OR "skilled") AND TI("training" OR "train*" OR "Learning" OR "learning" OR "learn*" OR "Education" OR "educat*" OR "Student" OR "practice")) **OR** (TI("Echography" OR "Ultrasound" OR "ultrasonography" OR "ultrasound" OR "ultras*" OR "sonography" OR "sonogra*" OR "echography" OR "echogra*" OR "echo") AND TI("Depth Perception" OR "Space Perception" OR "visuospatial skills" OR "visuospatial skill" OR "mental rotation" OR "perspective taking" OR "spatial perception" OR "Psychomotor Performance" OR "psychomotor skills" OR "psychomotor skill" OR "Motor Performance" OR "motor skills" OR "motor skill" OR "dexterity" OR "visuomotor skills" OR "visuomotor skill" OR "Vision" OR "anatomical knowledge" OR "anatomy knowledge" OR "physiological knowledge" OR "physiology knowledge" OR "Spatial orientation" OR "spatial skills" OR "spatial skill" OR "visuospatial abilities" OR "visuospatial ability" OR "psychomotor abilities" OR "psychomotor ability" OR "motor abilities" OR "motor ability" OR "visuomotor abilities" OR "visuomotor ability" OR "spatial abilities" OR "spatial ability" OR "Task Performance" OR "skills" OR "skill" OR "skilled") AND TI("training" OR "train*" OR "Learning" OR "learning" OR "learn*" OR "Education" OR "educat*" OR "Student" OR "practice")) **OR** TI(("point of care ultrasonography" OR "point of care ultrasound" OR "point of care ultras*" OR "POCUS" OR (("Point-of-Care Testing" OR "Point-of-Care Systems" OR "point of care" OR "point of car*" OR "pointofcare") AND ("Ultrasonography" OR "Ultrasonics" OR "ultrasonography" OR "ultrasound" OR "ultras*" OR "sonography" OR "sonogra*" OR "echography" OR "echogra*")) OR "bedside ultrasonography" OR "bedside ultrasound" OR "bedside ultras*") AND ("Depth Perception" OR "Space Perception" OR "visuospatial skills" OR "visuospatial skill" OR "mental rotation" OR "perspective taking" OR "spatial perception" OR "Psychomotor Performance" OR "psychomotor skills" OR "psychomotor skill" OR "Motor performance" OR "motor skills" OR "motor skill" OR "dexterity" OR "visuomotor skills" OR "visuomotor skill" OR "Vision" OR "anatomical knowledge" OR "anatomy knowledge" OR "physiological knowledge" OR "physiology knowledge" OR "Spatial orientation" OR "spatial skills" OR "spatial skill" OR "visuospatial abilities" OR "visuospatial ability" OR "psychomotor abilities" OR "psychomotor ability" OR "motor abilities" OR "motor ability" OR "visuomotor abilities" OR "visuomotor ability" OR "spatial abilities" OR "spatial ability" OR "Task Performance" OR "skills" OR "skill" OR "skilled") AND ("training" OR "train*" OR "Learning" OR "learning" OR "learn*" OR "Education" OR "educat*" OR "Student" OR "practice"))**)** NOT TI("veterinary" OR "rabbit" OR "rabbits" OR "animal" OR "animals" OR "mouse" OR "mice" OR "rodent" OR "rodents" OR "rat" OR "rats" OR "pig" OR "pigs" OR "porcine" OR "horse" OR "horses" OR "equine" OR "cow" OR "cows" OR "bovine" OR "goat" OR "goats" OR "sheep" OR "ovine" OR "canine" OR "dog" OR "dogs" OR "feline" OR "cat" OR "cats")**)**

**Appendix S2. Study characteristics**

| **Article** | **Number of participants** | **Description of participants** | **What was measured** | **Study design** | **Determinant test** | **Outcome test** | **Time elapsed** | **Analysis** | **Results** |
| --- | --- | --- | --- | --- | --- | --- | --- | --- | --- |
| **Knowledge** | |  |  |  |  |  |  |  |  |
| Baker et al. 2011^20^ | 25 | First and second year sonography students. | Relation between OSCE outcome and knowledge | Single group cross-sectional | Multiple choice test, questions unknown^¥^ | OSCE on standardized patient^1,2,3^ | Unknown | Pearson correlation between OSCE outcome and written test score | Weak correlation, r = 0.23 |
| Bell et al. 2016^21^ | 81 | Clinical officers, doctors, nurses, radiographers and one laboratory technician working in hospitals in Kenya. | Relation between knowledge and practical skill | Single group pretest and posttest | Written exam consisting of questions adapted from the ACEP Emergency Ultrasound Exam^1^ | ACEP emergency ultrasound exam. Rated by experts^1^ | 1 day - 7 months | Pearson correlation between written and practical test scores | Significant correlation, r = .44, *p* = < .01 |
| Berman et al.1986^22^ | 83 | Practicing sonographers | Relation between general reasoning score and sonography performance | Single group cross-sectional | Kit of Factor Referenced Cognitive Tests^1,3^ | SPES: sonographer performance evaluation scale^1^ | Unknown | Pearson correlation between SPES competence rating | Significant correlation, r = 0.345, *p* = ≤ .05 |
| Carrigan et al. 2020^24^ | 39 | 39 echocardiographers, 43 naïve participants | Relation between cue utilization and echocardiography performance | Non-randomized 2 groups | Expert Intensive Skills Evaluation platform (echocardiography edition)^1,2,3^ | Being naïve or an echocardiographer | Unknown | Difference in means between echocardiographers and naïve participants on cue utilization | Higher cue utilization associated with a greater mean number of years of experience (M = 12.51%, SD = 9.74) compared with lower cue utilization (M = 5.46%, SD = 9.06) |
| Chung et al. 2013^28^ | 49 | Medical and nursing students and a medical resident | Relation between performance measures and knowledge measures | Randomized controlled trial | Multiple-choice questionnaire on knowledge on FAST examination, abdominal anatomy, window interpretation^1,2^ | Diagnostic interpretation, acquisition of FAST window, window scan time^1^ | 285 minutes | Pearson correlation between performance measures and knowledge measures, diagnostic interpretation of FAST window and number of correct FAST window interpretations specifically | Significant association between 1 knowledge parameter and 1 performance parameter (r = 0.34, *p* = < .05) |
| Janjigian et al. 2021^35^ | 23 | Hospitalists | Relation between knowledge test scores and hands-on test scores | Single group pretest and posttest | Novel 20 item online knowledge test^1^ | Expert rated hands-on human examination by checklist^1^ | 2 days – 1 year | Pearson correlation between knowledge test scores and hands-on test scores | Highly correlated at one-year: r = .78, *p* = < .001 Moderate correlation on post-two-day assessment: r =.53, *p* = < .05 |
| Kissin et al. 2013^36^ | 38 | Rheumatology fellows | Relation between knowledge and practical skill | Non-randomized 2 groups | Multiple choice test^1,2,3^ | Expert rated practical examination^1,2,3^ | 21 hours | Pearson correlation between practical and written examination scores | Correlated in both groups: first group r = .70, *p* = < .001. Second group r = .59, *p* = < .05 |
| Nielsen et al. 2012^38^ | 73 | 28 third year medical students and 45 physicians | Relation between general physiology knowledge and TTE interpretation ability | Non-randomized 2 groups | Multiple choice test on cardiac physiology^1,2,3^ | TTE pathology checklist^1,3^ | Unknown | Spearman correlation between physiology test scores and TTE checklist scores | Significant relationship only found for residents: Spearman's rho = .78, *p* = < .001. No significant relationship between knowledge base and TTE score for interns (Spearman's rho = -0.35, *p* = .2) and consultants (Spearman's rho = .03, *p =* .91). |
| Schott et al. 2020^39^ | 30 | 21 MCCTP fellow and 9 advanced practice providers | Relation between baseline knowledge and baseline psychomotor skills | Single group pretest and posttest | Knowledge questionnaire^1^ | Standardized checklist of ultrasound^1,2^ | Academic year | Pearson correlation between baseline knowledge and baseline psychomotor scores | Significant correlation, r = .78, *p* = < .0001 |
| Shafqat et al. 2015^40^ | 66 | medical students with no previous experience with ultrasound | Relation between general cognitive ability and ultrasound task performance | Non-randomized 2 groups | Numerical Reasoning Test (NRT-20)^1,2,3^, Alice Heim Group Ability Test (AH4)^1,2,3^ | Expert rated GRS and CES^1,2,3^ | +/- 1 hour | Spearman correlation between Numerical Reasoning test and GRS and CES. | No significant correlation between NRT-20 and CES, Pearson correlation r=.01 p =.93 or GRS, r=-.05 p=.69). Or between AH4 and CES, r=-.09 p=.49 or GRS, r=.09, p=.0.49. |
| Sisley et al. 1999^41^ | 82 | 49 surgery and 33 emergency medicine physicians | Relation between factual knowledge and US interpretation ability | Non-randomized 2 groups | OSCE: written knowledge test (factual knowledge)^1,3^ | OSCE: image interpretation^1,3^ | Unknown (didactic + hands-on session) | Pearson correlation between knowledge test score and US interpretation examination scores | No significant correlation at precourse r = .20*, p* = > .05 and postcourse r = .14, *p* = > .05 |
| Stolz et al. 2018^43^ | 73 | Emergency medicine residents | Can knowledge be used as a predictor for performance on skills test? | Non-randomized 2 groups | Written knowledge assessment^1^ | OSCE^1^ | 2.5-4 hours | Proportion of variance in skills test than can be predicted by written pre-test scores | Written pre-test is not a good predictor: R2 = .028, *p* = .19 |
| Tolsgaard et al. 2019^44^ | 101 | Trainees of obstetrics and gynecology ultrasound | Relation between knowledge and diagnostic accuracy | Non-randomized 2 groups | Theoretical test^1,2,3,5^ | Diagnostic accuracy/OSAUS^1, 2, 3, 4^ | Unknown | Pearson correlation between theoretical test scores and diagnostic accuracy on simulator cases | No significant correlation between theoretical test scores and diagnostic accuracy. r = ?, *p* = .90 |
| Werner et al. 2016^46^ | 28 | PEM physicians | Relation between knowledge and pre-intervention competence | Single group pretest and posttest | Written knowledge test^¥^ | Direct observation US-guided CVC placement checklist^1,3^ | 2-12 months | Spearman correlation between preintervention performance and written examination score | Preintervention performance was positively correlated with written examination score: Spearman's rho = .39, *p* = < .05 |
| Woodworth et al. 2014^47^ | 65 | Residents and anesthesiologists | Relation between knowledge and scanning scores | Randomized controlled trial | Written questionnaire^1^ | Expert rated scanning assessment^1,2^ | 30 minutes | Pearson correlation between written test scores and scanning scores | Pretest correlation: r = .52, posttest correlation r = .64 |
| **Psychomotor ability** | |  |  |  |  |  |  |  |  |
| Chapman et al. 2016^26^ | 60 | 30 experienced sonographers (one year experience) and 30 trainees. | Relation between psychomotor ability and scanning ability | Randomized controlled trial | Perdue Pegboard test^1,2,3,4^, Zig-Zag Tracking test^1,2,3^ | Obstetric Structured Assessment Test (OSAT)^1,3^ | 12 months | Correlation between psychomotor test scores and post-training scanning scores. | No significant association between psychomotor scores and post-training scanning scores |
| Dromey et al. 2021^31^ | 20 | Novice and experienced obstetric sonographers | Difference in psychomotor ability between novice and expert groups | Non-randomized 2 groups | Dimensionless squared jerk (DSJ)^1,2,3^ | Being a novice or expert |  | Difference in DSJ scores between novice and expert group | Significant difference between Novice (M = 22.08, SD = 1.05) and experts (M = 19.26, SD = 3.02) *p =* .01 |
| Smith et al. 2012^42^ | 40 | Residents anesthesiology | Relation between psychomotor ability and scanning ability | Single group pretest and posttest | Projected Image Testing (Zig-Zag Test), Purdue Peg Board Test ^1,2,3,4^, Crawford Small Parts Dexterity Test^1,2,3^, Sennes-Weinstein Monofilament Sensory Testing^1,2,3^ | Composite performance score of expert rated simulator UGRA task^1^ | Unknown | Correlation between psychomotor test outcomes and ultrasound skill task outcome | No significant correlation between any of the psychomotor tests (Purdue Peg Board, Crawford's Small Parts Dexterity, Semmes-Weinstein Sensory) was found |
| Walker et al. 2019^45^ | 50 | Veterinary students with interest in radiology, but no experience. | Role of dexterity as a predictor for ultrasonographic and fluoroscopic skills in veterinary students | Non-randomized 2 groups | Grooved Pegboard test^1,2,3^, indirect Zig-Zag test^1,2,3^, 3D mouse proficiency test^1,2,3^ | Time to complete simulated ultrasound task^¥^ | Unknown | Correlation between dexterity test scores and ultrasound and fluoroscopic skill assessment scores | No significant association between dexterity scores and ultrasound assessment |
| **Visuospatial ability** | |  |  |  |  |  |  |  |  |
| Berman et al. 1986^22^ | 83 | Practicing sonographers | Relation between spatial orientation and flexibility of closure and ultrasound skill | Single group pretest and posttest | Kit of factor referenced cognitive tests: spatial orientation and visualization^1,3^ | SPES: sonographer performance evaluation scale^1^ | Unknown | Pearson correlation between spatial orientation score and self-reported competence | No significant correlation, r = .201, *p* = between .05 and .10 |
| Carrigan et al. 2020^24^ | 39 | 39 echocardiographers, 43 naïve participants | Relation between perceptual ability and NOMT accuracy | Non-randomized 2 groups | Novel Object Memory Test^1,2,3^ | Expert Intensive Skills Evaluation platform (echocardiography edition) ^1,2,3^ | Unknown | Difference in means between echocardiographers and naïve participants on NOMT and cue utilization score relation to NOMT | Echocardiographers scored better than naïve participants on NOMT t(38) = 2.96, *p* *=* .005 |
| Chapman et al. 2016^26^ | 60 | 30 experienced sonographers (one year experience) and 30 trainees. | Relation between visuospatial ability and scanning ability | Randomized controlled trial | Snowy Picture Test^1,2,3^, MRT-A^1,2,3^, Gestalt Completion Test^1,2,3^, The Surface Development Test^1,2,3^ | Obstetric Structured Assessment Test (OSAT)^1,3^ | 12 months | Correlation between visuospatial ability tests and post-training scanning performance | Lack of correlation between visuospatial ability test and post-training scanning performance. |
| Chuan et al. 2021^27^ | 140 | Medical students with no previous exposure to UGRA | Effect of visuospatial ability on UGRA learning | Randomized controlled trial | MRT-A^1,2,3^ | UGRA task^1,2,3^ | 20 minutes | Difference in GRS, time to complete task and final pass/fail rate for low and high visuospatial ability groups | No difference on time to complete task and GRS between groups. Low visuospatial ability group with no extra training had a significantly lower pass rate. |
| Clem et al. 2010^29^ | 17 | Sonography students, in cardiac, abdominal and ob/gyn | Relationship between visuospatial ability and scanning ability | Non-randomized 2 groups | Revised Minnesota Paper Form Board Test^1,2,3^ | Sonography Clinical Assessment Notebook ^1,3^ | Two semesters | Pearson correlation between visuospatial ability test score and scanning score after two semesters of instructions | r = .60, strong relationship between visuospatial ability and scanning ability. R2 = .36. |
| Clem et al. 2013^30^ | 79 | Beginners in cardiac, abdominal and ob/ gyn sonography students | Can spatial ability be used as a predictor for sonography skill acquisition? | Single group pretest and posttest | Revised Minnesota Paper Form Board Test^1, 2,3^ | Sonography Clinical Assessment Notebook^1,3^ | Two semesters | Pearson correlation between spatial pretest scores and scan scores. R2 for scanning competency with spatial pretest as a predictor. | Significant relationship between scanning and visuospatial ability after 30 hours of instruction r = .46, *p* = <.05, R2 = .21 and after two semesters of instruction r = .49, *p* = < .05, R2 = .23 |
| Duce et al. 2016^32^ | 33 | Ultrasound novices | Relation between visuospatial ability and sonography performance | Single group cross-sectional | MRT-A^1,2,3^, Shortened 14-item Concealed Figure Test^1,2,3^, Block Design Test^1,2,3^, Matrix Reasoning and Visual Puzzles^1,2,3^ | Expert rated sonography examination^1^ | 45 minutes | Spearman correlation between visuospatial ability test scores and sonography examination score. | Intermediate and high ability groups performed significantly better than low ability group (P < .02). Matrix Reasoning and Mental Rotation Test-A show significant correlation to final sonography exam score. MR: q = .38, *p* = .03, MRT-A: q = .36, *p* = .04 |
| Frederiksen et al. 2012^33^ | 21 | Physicians, no previous training in FATE or echocardiography and reference group of 3 examiners (>10 years’ experience) | Relation between visuospatial ability and image acquisition ability | Non-randomized 2 groups | Mental Rotation Test^1,2,3^ | FATE examination checklist^¥^ | 1 day | Pearson correlation between mental rotation test scores and global image rating and evaluation score. | Significant relationship between MRT and global image rating (r = .61, *p* = .003) and global image evaluation (r = .44, *p* = .047). Also moderate correlation between MRT scores and proper probe orientation (r = .56, *p* = .008) |
| Hewson et al. 2020^34^ | 94 | Undergraduate medicine, science, technology students no prior experience with UGRA or mental rotation | Effect of Mental Rotation training on ultrasound-guided regional anesthesia performance | Randomized controlled trial | MRT^1,2,3^ | Expert rated GRS^1,3^ and CES^1,3^ | 30 min | Effect size of mental rotation training on composite error score and global rating scale on post-training assessment | Large effect size of training on both composite error score (Cohen's d = .91, r = .41) and global rating scale (Cohen's d = .92, r = .42). Participants in the intervention made significantly fewer errors (*p* = < .001) and showed improved overall performance (*p* = .048) |
| Miller et al. 2017^37^ | 390 | First year medical students | Relation between visuospatial ability and sonography knowledge acquisition and US performance. | Single group cross-sectional | MRT^1,2,3^ | OSCE score^1^ |  | Pearson correlation between MRT and knowledge posttest and Pearson and Spearman correlation between MRT and OSCE scores | No significant relationship between MRT scores and OSCE scores, r = -.02, p =0.758. |
| Shafqat et al. 2015^40^ | 66 | Medical students with no previous experience with ultrasound | Relation between visuospatial ability and ultrasound task performance | Non-randomized 2 groups | MRT ^1,2,3^ | Expert rated GRS and CES^1,2,3^ | +/- 1 hour | Spearman correlation between mental rotation test scores and GRS and CES. | High CES scores (high error rate) was associated with low MRT scores (ρ = -0.54, *p* = < .001). Better GRS scores were associated with higher MRT scores (ρ = .47, *p* = .005). |
| Smith et al. 2012^42^ | 40 | Residents anesthesiology | Relation between visuospatial ability and ultrasound-guided procedure performance | Single group pretest and posttest | Block Design Test^1,2,3^, Digit Symbol Restitution Test^1,2,3^, Trailmaking Test^1,2,3^, Peli-Robison Contrast Acuity Test^1,2,3^ | Composite performance score of expert rated simulator UGRA task^1^ | Unknown | Correlation between visuospatial assessment scores and global ultrasound performance (composite Z scores) | The Block Design Test significantly correlated with global ultrasound performance (r = .47, *p* = <. 01). Block design test also positively correlated with Indirect Zig-Zag test performance (r = .41, *p* = < .01) |
| Walker et al. 2019^45^ | 50 | Veterinary students with interest in radiology, but no experience. | Role of visuospatial ability as a predictor for ultrasonographic and fluoroscopic skills in veterinary students | Non-randomized 2 groups | Purdue visualization of rotation test, MRT^1,2,3^, Raven's advanced progressive matrices test ^1,2,3^ | Time to complete simulated ultrasound task^¥^ | Unknown | Correlation between visuospatial ability test scores and ultrasound and fluoroscopic skill assessment scores | Significant positive association between MRT scores and time to complete cystocentesis task (*p =* .001). Significant association between MRT scores and time to complete endovascular fluoroscopy task (*p = .*029) |

**GRS = Global Rating Scale, CES = Composite Error Scores, MRT = Mental Rotation Test, MRT-A = Revised Vanderberg and Kruse mental rotations test-A , OSCE = Objective Structured Clinical Examination, OSAT = Obstetric Structured Assessment Test, OSAUS = Objective Structured Assessment of Ultrasound, SPES: Sonographer Performance Evaluation Scale, UGRA = Ultrasound Guided Regional Anesthesia, US = ultrasound.**

**Validity evidence of the measurement/assessment according to the Messick framework^30^:**

**^1^ Content =** The relationship between the content of a test and the construct it is intended to measure

**^2^ Internal structure =** Relationship among data items within the assessment and how these relate to the overarching construct

**^3^ Relationships with other variables =** Degree to which these relationships are consistent with the construct underlying the proposed test score interpretations

**^4^ Response process =** The fit between the construct and the detailed nature of performance . . . actually engaged in

**^5^ Consequences =** The impact, beneficial or harmful and intended or unintended, of assessment.

**^¥^ unknown**

**Appendix S3. MERSQI table**

| **Study** | **Study design** | **Sampling: institutions** | **Sampling: response rate** | **Type of data** | **Validity evidence for evaluation instrument score** | **Data analysis: sophistication** | **Data analysis: appropriate** | **Outcome** | **MERSQI sum score** |
| --- | --- | --- | --- | --- | --- | --- | --- | --- | --- |
| Baker et al. 2011 | 1 | 0.5 | 1.5 | 3 | 3 | 2 | 1 | 1.5 | 13.5 |
| Bell et al. 2016 | 1.5 | 1.5 | 0.5 | 3 | 1 | 2 | 1 | 1.5 | 12 |
| Berman et al. 1986 | 1 | 0.5 | 1.5 | 3 | 1 | 2 | 1 | 1.5 | 11.5 |
| Carrigan et al. 2020 | 2 | 1.5 | 1.5 | 3 | 3 | 2 | 1 | 1.5 | 15.5 |
| Chapman et al. 2016 | 3 | 0.5 | 1.5 | 3 | 1 | 2 | 1 | 1.5 | 13.5 |
| Chuan et al. 2021 | 3 | 1.5 | 1.5 | 3 | 1 | 2 | 1 | 1.5 | 14.5 |
| Chung et al. 2013 | 3 | 0.5 | 1.5 | 3 | 2 | 2 | 1 | 1.5 | 14.5 |
| Clem et al. 2010 | 2 | 0.5 | 1.5 | 3 | 1 | 2 | 1 | 1.5 | 12.5 |
| Clem et al. 2013 | 1.5 | 1.5 | 1.5 | 3 | 1 | 2 | 1 | 1.5 | 13 |
| Dromey et al. 2021 | 2 | 0.5 | NA | 3 | 0 | 2 | 1 | 1.5 | 10 |
| Duce et al. 2016 | 1 | 0.5 | 1.5 | 3 | 1 | 2 | 1 | 1.5 | 11.5 |
| Frederiksen et al. 2012 | 2 | 0.5 | 1.5 | 3 | 1 | 2 | 1 | 1.5 | 12.5 |
| Hewson et al. 2020 | 3 | 0.5 | 1.5 | 3 | 2 | 2 | 1 | 1.5 | 15.5 |
| Janjigian et al. 2021 | 1.5 | 1.5 | 1.5 | 3 | 1 | 2 | 1 | 1.5 | 13 |
| Kissin et al. 2013 | 2 | 1.5 | 0.5-1 | 3 | 2 | 2 | 1 | 1.5 | 12.5-13 |
| Miller et al. 2017 | 1 | 0.5 | 1.5 | 3 | 1 | 2 | 1 | 1.5 | 11.5 |
| Nielsen et al. 2012 | 2 | 1.5 | 1.5 | 3 | 3 | 2 | 1 | 1.5 | 15.5 |
| Schott et al. 2020 | 1.5 | 0.5 | 1 | 3 | 2 | 2 | 1 | 1.5 | 12.5 |
| Shafqat et al. 2015 | 2 | 0.5 | 1 | 3 | 3 | 2 | 1 | 1.5 | 14 |
| Sisley et al. 1999 | 2 | 0.5 | 1 | 3 | 1 | 2 | 1 | 1.5 | 12 |
| Smith et al. 2012 | 1.5 | 0.5 | 1.5 | 3 | 3 | 2 | 1 | 1.5 | 14 |
| Stolz et al. 2018 | 2 | 1.5 | 1.5 | 3 | 3 | 2 | 1 | 1.5 | 13.5 |
| Tolsgaard et al. 2019 | 2 | 1.5 | 1.5 | 3 | 1 | 2 | 1 | 1.5 | 14 |
| Walker et al. 2019 | 2 | 0.5 | 1.5 | 3 | 1 | 2 | 1 | 1.5 | 12.5 |
| Werner et al. 2016 | 1.5 | 0.5 | 1.5 | 3 | 2 | 2 | 1 | 1.5 | 13 |
| Woodworth et al. 2014 | 3 | 1 | 1.5 | 3 | 2 | 2 | 1 | 1.5 | 15 |

**Appendix S4. Aptitude tests**

| **Tests** | **Used to measure** | **Relevant articles** | **Correlations in the literature** | **Test description** |
| --- | --- | --- | --- | --- |
| **Relevant knowledge** |  |  |  |  |
| Image interpretation |  |  |  |  |
| ACEP emergency ultrasound examination | Anatomical knowledge, pathology recognition, clinical reasoning | Bell et al. 2016 | Strong significant correlation between theoretical knowledge and practical skill assessment outcome | Multiple choice questionnaire consisting of video, image and case-based questions |
| Pretest Chung et al. 2013 | Prior knowledge of abdominal anatomy, basic FAST examination procedures and window interpretation. | Chung et al. 2013 | Significant correlation between diagnostic interpretation of FAST window and number of correct FAST window interpretations and ultrasound performance window interpretations. No significant correlation between anatomical knowledge and ultrasound performance measures. | 69-item pretest related to FAST examination. FAST examination (15 constructed response items and 46 selected response items) and prior knowledge of abdominal anatomy (8 selected response items). |
| Online test Janjigian et al. 2020 | Basic ultrasound knowledge, image interpretation and clinical interpretation. | Janjigian et al. 2020 | Significant correlation between knowledge test scores and hands-on test scores | Novel 20-item online test, basic ultrasound knowledge, image interpretation and clinical interpretation. |
| Multiple-Choice Question Examination Kissin et al. 2013 | Knowledge of rheumatologic musculoskeletal ultrasound pathology, image interpretation | Kissin et al. 2013 | Significant correlation between practical and written test scores | 107 multiple-choice questions rheumatologic musculoskeletal ultrasound images for interpretation |
| Physiology knowledge test and TTE interpretation checklist Nielsen et al. 2012 | Basic cardiac physiology and pathology recognition | Nielsen et al. 2012 | Significant correlation between physiology test scores and pathology recognition in residents | Basic knowledge about cardiac physics. Recognition of pathology in shown echocardiography images |
| Multiple choice quiz Schott et al. 2020 | Knowledge about ultrasound physics, device knobology, image interpretation for identification of anatomical structures and pathology and therapeutic decision making. | Schott et al. 2020 | Significant correlation between baseline knowledge and baseline psychomotor scores | 30-question online multiple-choice quiz about ultrasound physics, device knobology and image interpretation for identification of anatomical structures and pathology and therapeutic decision making. |
| OSCE written knowledge test | Factual ultrasound knowledge & clinical interpretation skills | Sisley et al. 1999 | Significant correlation between knowledge test score and US interpretation examination scores | Multiple-choice questionnaire factual knowledge of US physics, US technology, clinical applications. US interpretation during clinical scenarios. |
| Written knowledge assessment Stolz et al. 2018 | Basic ultrasound physics, system workflow, anatomy and image interpretation | Stolz et al. 2018 | Written pre-test is not a good predictor of ultrasound performance | 37-questions including basic ultrasound physics, system workflow, anatomy and image interpretation (artifacts, pathology, and appropriate ultrasound settings) |
| Written test Woodworth et al. 2014 | Image interpretation: anatomical knowledge | Woodworth et al. 2014 | Significant correlation between written test scores and scanning scores | 30-question written test on identification of anatomic structures on US images |
| Technical aspects |  |  |  |  |
| Online test Janjigian et al. 2020 | Basic ultrasound knowledge, image interpretation and clinical interpretation. | Janjigian et al. 2020 | Significant correlation between knowledge test scores and hands-on test scores | Novel 20-item online test, basic ultrasound knowledge, image interpretation and clinical interpretation. |
| Multiple choice quiz Schott et al 2020 | Knowledge about ultrasound physics, device knobology, image interpretation for identification of anatomical structures and pathology and therapeutic decision making. | Schott et al. 2020 | Significant correlation between baseline knowledge and baseline psychomotor scores | 30-question online multiple-choice quiz about ultrasound physics, device knobology and image interpretation for identification of anatomical structures and pathology and therapeutic decision making. |
| OSCE written knowledge test | Factual ultrasound knowledge & clinical interpretation skills | Sisley et al. 1999 | Significant correlation between knowledge test score and US interpretation examination scores | Multiple-choice questionnaire factual knowledge of US physics, US technology, clinical applications. US interpretation during clinical scenarios. |
| Written knowledge assessment Stolz et al. 2018 | Basic ultrasound physics, system workflow, anatomy and image interpretation | Stolz et al. 2018 | Written pre-test is not a good predictor of ultrasound performance | 37-questions including basic ultrasound physics, system workflow, anatomy and image interpretation (artifacts, pathology, and appropriate ultrasound settings) |
| Written examination Werner et al. 2016 | Knowledge about use of US for CVC placement | Werner et al. 2016 | Significant correlation between preintervention performance and written examination score | 20-question computerized examination about knowledge around the use of US for CVC placement |
| General cognitive ability |  |  |  |  |
| Kit of Factor Reference Cognitive Test | General reasoning | Berman et al. 1986 | Significant correlation between SPES competence rating and GRS | Participants perform tasks of pattern recognition, visual manipulation and picking out an object from an interfering background |
| EXPERTise 2.0 Echocardiography edition | Cue utilization | Carrigan et al. 2020 | Significant relationship between cue utilization and self-reported years of experience | Feature identification task, feature recognition task, feature association task, feature discrimination task and a feature prioritization task |
| Alice Heim Group Ability Test | Verbal, mathematical and spatial reasoning | Shafqat et al. 2015 | No significant correlation found | Participant completes 130 items regarding series completion, mental arithmetic, vocabulary, and reasoning |
| Numerical Reasoning Test (NRT-20) | Fluid intelligence, abstract reasoning, problem-solving | Shafqat et al. 2015 | No significant correlation found | Participant has to solve 20 short reasoning problems based on numbers |
| **Psychomotor Skills** |  |  |  |  |
| Crawford small parts dexterity test | Dexterity | Smith et al. 2012 | No significant correlation found | Participant is timed while placing small pins and screws in holes in a plate. Scored by amount of plates completed or time taken to complete task |
| Digit symbol substitution test* | Attention, visual scanning, dexterity, speed and coordination | Smith et al. 2012 | No significant correlation found | Participant has to pair symbols to numbers and fill in the appropriate symbol below a shown number while being timed |
| Dimensionless Squared Jerk | Efficiency of movement | Dromey et al. 2021 | Significant difference between novice and expert ultrasound users. | Efficiency of movement is assessed using a formula which uses movement amplitude or extent, initial time, final time, completion time, pathlength and duration |
| Projected image test (zigzag test) | Dexterity | Smith et al. 2012, Chapman et al. 2016,Walker et al. 2019 | Smith: indirect Zig-Zag test significantly correlated with The Block Design Test | Maze completion on table and on projection with both hands, to test both direct and indirect hand-eye coordination |
| Purdue pegboard test | Dexterity for gross movements of arms, hands and fingers & fingertip dexterity | Smith et al. 2012, Chapman et al. 2016,Walker et al. 2019 | No significant correlation found | Timed measure of placing pegs in a board to measure coordination, speed and dexterity |
| Semmes-weinstein monofilament sensory testing | Tactile perception | Smith et al. 2012 | No significant correlation found | Sensory perception test using monofilaments on the fingers and hand to test tactile sensation sensitivity |
| **Visuospatial Manipulation** |  |  |  |  |
| Visualization |  |  |  |  |
| Kit of Factor Reference Cognitive Tests (spatial orientation and visualization subtests) | Object recognition, visualization and flexibility of closure | Berman et al. 1986 | No significant correlation found | Participant has to identify one of five simple figures in a complex figure |
| Revised Minnesota Paper Form Board Test | Spatial visualization, part-whole relationships | Clem et al. 2010, Clem et al. 2013 | Clem 2010: Significant association between scanning ability and visuospatial ability; Clem 2013: significant association between scanning ability and visuospatial ability after 30 hours of instruction and after full program | Participant has to decide which out of 5 figures best represents a combination of shown pieces |
| Surface Development Test | High level visuospatial ability, visualization | Chapman et al. 2016 | No significant correlation found | Participants have to visualize how a 2d image can be folded into a 3d structure. Participants pairs numbers and letters based on how they think the 3d structure is formed from the template |
| (Speeded) Mental Rotation |  |  |  |  |
| Mental Rotation Test | High level visuospatial ability, spatial relations | Chapman et al. 2016, Hewson et al. 2020, Shafqat et al. 2015, Walker et al. 2019 | Chapman: no significant relationship; Hewson: Participants with MRT training made significantly fewer errors and showed significantly improved overall performance, Shafqat: High error rate was significantly correlated with low MRT score and low error rate was significantly correlated with high MRT score; Walker: Significant association between MRT score and task completion time | Participant has to mentally manipulate a shown image to establish which are and are not possible rotations of the shown image |
| Revised Vanderberg and Kruse Mental Rotation Test A | High level visuospatial ability, spatial relations | Chuan et al. 2021,Duce et al. 2016, Frederiksen et al. 2012, Miller et al. 2017 | Chuan: Low visuospatial ability group with no extra training had significantly lower passing rate; Duce: Intermediate and high ability groups performed significantly better than low ability group and MRT shows significant correlation to final sonography exam score; Frederiksen: Significant relationship between MRT and global image rating and evaluation also significant correlation between MRT and proper probe orientation; Miller: No significant relationship between MRT scores and US performance | Participant has to mentally manipulate a shown image to establish which are and are not possible rotations of the shown image |
| **Visuospatial Perception** |  |  |  |  |
| Lower order perception |  |  |  |  |
| Pelli-Robson contrast acuity testing | Ability to distinguish between different shades of gray | Smith et al. 2012 | No significant correlation found | Participant has to read as many letters as possible, while they become increasingly less visible |
| Closure speed |  |  |  |  |
| Block Design Test (WAIS-IV) | Spatial visualization and closure speed | Duce et al. 2016, Smith et al. 2012 | Duce: Intermediate and high ability groups performed significantly better; Smith: The Block Design Test significantly correlated with global ultrasound performance. Block Design Test was also positively correlated with indirect Zig-Zag test performance. | Two-dimensional views of a three-dimensional design are shown. The participant is asked to recreate the design using coloured wooden blocks, under time constraints |
| Matrix reasoning Test | Visual processing, abstract spatial perception | Duce et al. 2016 | Intermediate and high ability groups performed significantly better, significant correlation to final sonography exam score | Participant has to logically conclude the next figure in a sequence based on pattern recognition |
| The Snowy Picture Test | Intermediate level visuospatial ability, speed of closure | Chapman et al. 2016 | No significant correlation found | Participants are shown a grey interference picture. Pattern recognition in the interference implies illusionary pattern recognition. |
| Visual puzzles | Nonverbal reasoning, analyze and synthesize abstract visual stimuli | Duce et al. 2016 | Intermediate and high ability groups performed significantly better | Participant are required to pick pieces which together form a shown image |
| Flexibility of closure |  |  |  |  |
| Abstract Reasoning Test | High level visuospatial ability, visual analysis, general reasoning skills | Chapman et al. 2016 | No significant correlation found | Non-verbal reasoning test requiring participant to form hypotheses, identify and apply patterns in diagrammatic form |
| Concealed Figures Test | Perceptual flexibility | Duce et al. 2016 | Intermediate and high ability groups performed significantly better | Participant is asked to identify a simple figure in multiple complex figures |
| Group Embedded Figures Test | Field (in)dependence | Shafqat et al. 2015 | No significant correlation found | Participant has to identify a simple form in 18 complex figures |
| Kit of Factor Reference Cognitive Tests (flexibility of closure subtest) | Object recognition, visualization and flexibility of closure | Berman et al. 1986 | No significant correlation found | Participant has to identify one of five simple figures in a complex figure |
| Visual memory |  |  |  |  |
| Novel Object Memory Test | Object recognition | Carrigan et al. 2020 | Significant better score on NOMT for echocardiographers compared to naïve participants | Participant has to memorize novel objects. Novel objects are used to make sure there is no 1 rule that can be applied to memorize all categories |
| Spatial scanning |  |  |  |  |
| Trail making test (TMT) | Visual-motor skills | Smith et al. 2012 | No significant correlation found | Participants are timed while using a pen or pencil to connect dots based on numerical, alphabetical, or mixed order |
|  |  |  |  |  |
